# Unveiling Clutch Investment Strategies in Birds: A Case Study on Great Tits Using Generalized Additive Models

**DOI:** 10.1002/ece3.71700

**Published:** 2025-07-01

**Authors:** Lluís Socias‐Martínez, Elena Álvarez, Louise R. Peckre, Emilio Barba

**Affiliations:** ^1^ Department of Sciences and Technology, Institut National Universitaire Jean‐François Champollion The University of Toulouse Albi France; ^2^ “Cavanilles” Institute of Biodiversity and Evolutionary Biology University of Valencia Valencia Spain; ^3^ Laboratoire d'Ethologie Expérimentale et Comparée UR 4443 (LEEC) Université Sorbonne Paris Nord Villetaneuse France

**Keywords:** brood reduction strategy, brood survival strategy, egg laying order, egg volume, non‐linear modeling, *Parus major*, reproductive strategy, temporal autocorrelation

## Abstract

Reproductive effort involves trade‐offs among offspring and with homeostasis. In birds, a crucial parental investment concerns the allocation of resources to each egg. Variation in egg investment has led to the development of hypotheses regarding whether females favor the eldest nestlings (“brood reduction”) or distribute resources more evenly (“brood survival”). Alternative explanations include the maturation of reproductive organs and trade‐offs with environmental constraints, such as thermoregulation. The earliest tests of these hypotheses relied on the deviation of the last egg's volume from the clutch mean (*D*). More recent studies have often overlooked non‐linearity and temporal autocorrelation in egg investments. Here, we analyze 145 broods comprising 1084 eggs from a great tit ( *Parus major* ) population in eastern Spain using generalized additive mixed‐effects models (GAMMs). Laying order influenced egg volume, with most variation occurring as an increase from the first to middle eggs in the clutch, followed by a slight decrease. We favor the interpretation that females adopt a brood survival strategy because (1) volume changes were of small magnitude (approx. 5%); (2) no significant negative effects of the number of eggs developing simultaneously in the ovary, total clutch size, or minimum temperatures were found; and (3) the laying order effect prevailed throughout the breeding season. However, within this brood survival strategy, females slightly favored middle eggs. In addition, negative correlations among deviations in volume from the main strategy suggest that energetic trade‐offs were present. Finally, most variation occurred between clutches, reinforcing previous findings of limited plasticity in egg volume in birds. Our results further caution against relying on mean egg volume (*D*) as a measure of investment strategies and highlight the importance of considering non‐linearity and temporal autocorrelation in analyses of reproductive investment. The use of GAMMs provides a robust approach to overcoming previous analytical limitations in the study of within‐clutch variation in egg investment.

## Graphical abstract

Reproductive investment in eggs can be made egalitarian or unevenly distributed within clutches. We investigated the pattern of investment in great tits ( *Parus major* ) from a Spanish population using generalized additive mixed‐effects models. Our results indicate that females consistently invest less in the first eggs.

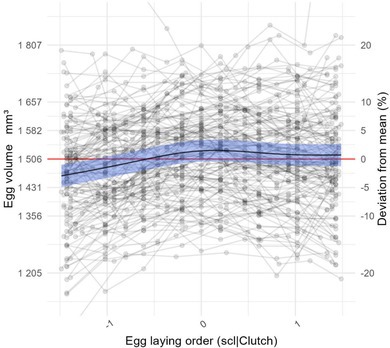

## 1 Introduction

Life‐history theory predicts that reproductive investments will be traded off against other energetic expenditures (Stearns 1992). One of the most salient expectations arising from investment trade‐offs is the negative relationship between offspring number and quality (Clutton‐Brock 1991; Stearns 1992; Royle et al. 2012). This negative correlation has been observed across taxa (Fleming and Gross 1990; Blackburn 1991; Christians 2000; Williams 2001; Charnov and Ernest 2006; Song et al. 2020). In addition, trade‐offs in energetic or temporal investments occur both between and within breeding attempts. Parents might be selected to adopt reproductive strategies that maximize lifetime reproductive success by adjusting investment in different broods or offspring depending on expected payoffs. Consequently, investment in offspring number, quality, and sex should be conditional on internal and external factors as well as on future reproductive opportunities within the same or subsequent breeding periods (Trivers and Willard 1973; Slagsvold et al. 1984; Stearns 1992).

Egg volume has been linked to offspring survival and fecundity in birds (Slagsvold et al. 1984; Williams 1994; Krist 2011), making it a key component of maternal investment. As predicted by life‐history theory, clutch size tends to be negatively correlated with egg size across avian species (Blackburn 1991; Christians 2000; Williams 2001; Song et al. 2020). Within bird populations, most variation in egg volume is explained by female identity, and egg size is highly repeatable and heritable compared to other reproductive traits (Christians 2002). Thus, egg volume has been suggested to be relatively fixed around an optimal value, with the number of offspring per brood and the timing of laying being the primary variables for adjustment in response to internal (e.g., body condition) and external (e.g., temperature) variation (Christians 2002). Despite this proposed stability, evidence suggests that females adjust the egg volume within clutches. You et al. (2009) identified three main patterns of egg volume variation as a function of laying order: an increase, a decrease, or an increase followed by a decrease.

Several hypotheses have been proposed to explain these patterns based on maternal manipulation of offspring survival and competitiveness through variation in egg size. The increasing pattern is thought to reflect a strategy that maximizes the survival of each offspring in the face of asynchronous hatching (the “brood survival hypothesis”; Clark and Wilson 1981; Slagsvold et al. 1984). In this case, mothers compensate for the development disadvantages of later‐hatched chicks by increasing egg size (Howe 1976). The second pattern, a decrease in egg size as a function of laying order, has been associated with a differential investment strategy favoring earlier offspring (the “brood reduction hypothesis”; Edwards Jr. and Collopy 1983; Viñuela 1997). This strategy is thought to be associated with producing more offspring than the environment can support, with surplus offspring being smaller and only successfully reared under particularly favorable conditions. The third pattern, an increase followed by a decrease, could reflect a mixed strategy, where females initially follow a “brood survival” strategy but later produce smaller, less costly eggs in anticipation of variable environmental conditions. Additionally, some species may switch between these strategies depending on external or internal factors (e.g., Christensen and Balsby 2020).

Alternative hypotheses suggest that variation in egg volume is primarily driven by female condition at the time of egg production. According to such hypotheses, if females had equal investment capacities throughout laying, we would expect a constant mean egg volume with random variation. However, constraints on female physiology may lead to systematic variation. First, reproductive organs may not maintain consistent efficiency, resulting in changes in egg volume as laying progresses (Parsons 1976). For instance, if ovarian maturation is not complete at the beginning of egg laying, egg volume might increase with laying order as a direct consequence of improving ovarian efficiency. Second, egg volume may be influenced by fluctuations in female energy levels (Järvinen and Ylimaunu 1986; Wiebe and Bortolotti 1996). In highly seasonal environments, the first eggs may be produced under conditions of food scarcity and/or low temperatures that increase thermoregulatory costs, leading to a subsequent increase in egg volume as conditions improve. Both effects could produce an increase in egg volume without needing to invoke a “brood survival” strategy. Conversely, if investment in earlier eggs depletes maternal energy reserves, a carryover effect could cause a decline in egg volume with laying order without the need to appeal to a “brood reduction” strategy. The third pattern of increase and decrease could likewise occur by a combination of the above‐mentioned effects arising from female condition.

Previous studies examining clutch investment strategies in birds have suffered from three limitations: they (1) relied on diverse forms of data simplification, (2) ignored temporal autocorrelation, and (3) did not test systematically for competing hypotheses. Regarding (1), the broadest and most cited comparative study to date on egg size variation as a function of laying order focuses on a single egg. Slagsvold et al. (1984) analyzed the deviation of the final egg relative to the mean clutch volume (denoted as *D*) across various avian orders as a function of several life history and ecological variables. The authors justified their choice because they found that the overall level of change between one egg and the next was highly correlated to *D* in the Hooded Crow (*r* = 0.55, *N*_clutch_ = 16). Despite the advantages of such simple and broadly applicable measure, *D* accurately describes investment strategies whenever the last egg is the only one to consistently deviate from the mean or when the progression in egg volume is monotonic and/or linear. Recent studies using the entire clutch, however, suggest that variation in egg volume arises through deviations in all eggs in a non‐linear way (Table 1). These studies have dealt with non‐linearity by using quadratic terms or transforming egg position into a factor within linear mixed models or ANOVAs. Regarding (2), while some of the recent studies consider all eggs in a clutch using linear mixed‐effects models, a method that can explicitly consider investment dependence across eggs within clutches using time series has not yet been applied (Table 1). Regarding (3), most studies did not aim at testing the brood reduction or survival hypotheses nor alternative hypotheses based on female condition. Because of this, other sources of variation in egg volume, such as trade‐offs with thermoregulation, have yet to be tested (Table 1). New studies could benefit from using a multivariate non‐linear modeling framework accounting for temporal autocorrelation.

**TABLE 1:** Studies found analyzing or reporting egg volume or mass as a function of laying order Columns “Order” indicates the taxonomic order of the species; Species gather the common and scientific names of species; Location, the country where the study was carried out, *N*_f_”, the number of females included in the analyses; “*N*_b_”, the number of clutches; “*N*_e_”, the number of eggs; “Measure”, whether mass or volume was used to characterize eggs; “Pattern”, a graphical simplified representation of the patterns of change (“/” indicating increase, “\” decrease, “_” no change); “NL”, the presence “1” or absence “0” of non‐linearity in the data; “Description”, a simplified description of the results complementing the two previous columns; “Max diff”, the difference in the mean predicted measure expressed as a percentage of the mean between the most different egg laying orders; “Methodology”, the method used to test for and assess the existence, magnitude, and direction of differences; “Covariates”, those variables used in combination with egg laying order to explain egg mass or volume; “Figure”, the number of the figure in the study displaying the results; and “Study”, the names and year of publication of the study containing the information presented in the other columns.

Order	Species	Location	*N*_f_	*N*_c_	*N*_e_	Measure	Pattern	NL	Description	Max diff	Methodology	Covariates	Figure	Study
Anseriformes	Common eiders ( *Somateria mollissima* )	Denmark	812	1099	4531	Volume	\ and /\	1	Pattern depends on clutch size. Small clutches pattern 1, then as clutch size increases, pattern 3 and for biggest clutch sizes pattern 2 with a plateau at the beginning	Largest clutches 10%	Repeated measures ANOVA	Clutch size, Year, Clutch ID	2	(Christensen and Balsby 2020)
Psittaciformes	Budgerigars ( *Melopsittacus undulatus* )	Laboratory	36	36	211	Mass	/\/	1	Second egg is the heaviest, sixth lightest and the others intermediate	?	GLMM	Female weight, Mate preference, Clutch order, Clutch size, Clutch and Female ID	5A	(Lahaye et al. 2015)
Passeriformes	Spotless starlings ( *Sturnus unicolor* )	Spain	319	647	2885	Volume	\	1	First and second eggs largest then gradual decrease	2.50%	GLMM with quadratic term	Female ID	2	(Monclús et al. 2017)
Passeriformes	Pied flycatcher ( *Ficedula hypoleuca* )	Spain	106	106	594	Volume	\/	1	First and last eggs are largest, decrease followed by an increase	18%	LMM with quadratic term	Female weight, F age, Laying date, Clutch size, Female ID	2	(Fuertes‐Recuero et al. 2025)
Passeriformes	Blue tit (*Parus caeruleus*)	Sweden	53	53	—	Mass	/_/	1	First egg is smallest and later biggest if asynchronously incubated	4%	Correlation and Paired‐sample *T*‐test	—	3	(Nilsson and Svensson 1993)
Passeriformes	Great tit ( *Parus major* )	Belgium	8	8	47	Mass	\_\	1	First egg is the largest, last smallest, others intermediate	Approx. 75%	LMM (as ANOVA) + Tukey post hoc	Clutch ID	—	(Lasters et al. 2019)
Passeriformes	Great tit ( *Parus major* )	Belgium	8	8	47	Volume	_	0	No significant difference	—	LMM (as ANOVA) + Tukey post hoc	Clutch ID	5	(Lasters et al. 2019)
Passeriformes	Great tit ( *Parus major* )	The Netherlands	—	104	741	Mass	\/	1	Reported “[egg mass] initially decreased and then increased through the laying sequence”	—	—	—	—	(Lessells et al. 2002)
Passeriformes	Great tit ( *Parus major* )	Estonia	15	15	30	Volume	_	—	Only first and last eggs compared	—	Differencing last and first eggs	—	1	(Hõrak et al. 2002)
Passeriformes	Great tit ( *Parus major* )	Estonia	15	15	30	Mass	/	—	Only yolk first and last eggs compared	—	Differencing last and first eggs	—	1	(Hõrak et al. 2002)
Passeriformes	Great tit ( *Parus major* )	North‐eastern China	—	30	337	Volume	/	1	Increase in volume	Approx. 5%–10%	One‐way ANOVA	—	2	(You et al. 2009)
Passeriformes	Great tit ( *Parus major* )	North‐eastern China	—	30	337	Mass	/	1	Increase in mass	—	One‐way ANOVA +LM	—	2	(You et al. 2009)

The use of hierarchical generalized additive mixed‐effects models (GAMMs hereafter; Wood 2017; Pedersen et al. 2019) could prove useful in this context. GAMMs can model complex non‐linear functions without constraining their shape before model fitting and can incorporate multivariate effects and sources of dependence, such as random effects. We take advantage of GAMMs to explore clutch investment strategies in light of existing hypotheses in a great tit ( *Parus major* ) population from south‐eastern Spain. Previous studies investigating egg variation in relation to laying order in *Parus major* found a multitude of relationships, perhaps suffering from small sample sizes and the three methodological limitations reviewed in the previous paragraph (Table 1). Given the taxonomic variation in patterns of egg volume across the laying order, the observed within‐species diversity, and the limitations found, our study can contribute both valuable data for future comparative analyses of this biological phenomenon and a new methodological approach.

Specifically, we test for variation in egg volume arising from (1) an effect of laying order, and we predict an increase in egg volume if females use a brood survival strategy or undergo a maturation of ovarian tissue during laying, a decrease if they follow instead a brood reduction strategy or there is a carryover of costs of egg production during egg laying, or a non‐linear change if females follow a mixed strategy or suffer from maturation and carryover effects; (2) a changing effect of laying order depending on clutch size, and we predict that as clutch size increases, last eggs become relatively smaller if females follow a brood reduction or suffer from increased costs when under conditions of high competition within clutches; (3) a negative effect of temperature on egg volume, and we predict that as temperatures lower, egg size decreases if female reproductive investment is traded off against thermoregulation; and (4) a dependence of investments in egg volume between consecutive eggs, and we predict that after accounting for main strategies of egg volume as a function of laying order, autocorrelation should be negative if egg volume investments are traded off against each other.

## 2 Materials and Methods

### 2.1 Study Population and Measures

The great tit ( *Parus major* ) is a small passerine bird from the family Paridae, with an average mass of 17–19 g and a wingspan of 22–25 cm in Spanish populations (Atiénzar et al. 2016). Our study population was in Sagunto, Valencia (eastern Spain, 39°42′ N, 0°15′ W, 30 m a.s.l.), within an area dominated by orange monoculture. The population has been monitored since 1986 using artificial nest boxes (Álvarez and Barba 2014; Solís et al. 2021), but individual identification of adult birds was not available in the early years when the data of the present study was collected (1988, 1990, 1992, 1993, and 1994). Great tits typically lay one egg per day during the laying period until the clutch is complete. Each egg develops in the oviduct for 3 days before being laid shortly after sunrise on the fourth day (Song et al. 2020). In this population, females may produce up to two broods per year, although most laid only one during the years of this study (Solís et al. 2021). During the years when the data used in this study was collected, clutch size was 7.68 ± 0.67 (mean ± std. deviation, *N*_years_ = 5).

For this study, a subset of randomly selected nests was checked daily from the beginning of nest construction during each breeding season. Once egg laying began, each egg was individually marked with a consecutive number using a permanent felt pen. When the clutch was completed (no new eggs were found for two consecutive days), the length and breadth of each egg were measured with a caliper. All measurements were taken by the same observer (E.B.) using the same caliper across the five study years to ensure consistency.

The egg volume was estimated using the formula: $v=0.4673LB^{2}+0.042$, where *L* is the length, *B* is the breadth, and the constants were specifically estimated for great tits (Ojanen et al. 1978). Egg sphericity was calculated as a ratio between width and length (width/length; Hõrak et al. 1995). During the study period, some pairs laid a second clutch (after a successful first one) or a replacement clutch (after failure). As it is standard in these studies (e.g., Van Noordwijk et al. 1995; Solís et al. 2023), we considered as “first” clutches those that started within the 30 days following the start of the first clutch each year, classifying the rest as “late” clutches, which might include both replacement and second clutches. In total, 1084 eggs from 145 different clutches were measured over the 5 breeding seasons.

As, by the time of egg laying, low temperatures are expected to act as a constraint, minimum temperatures for the 3 days preceding each egg's laying date (i.e., the period of egg formation) were obtained from a nearby (c.a. 3 km) meteorological station operated by the Spanish Meteorological Agency (AEMET).

The authorization to carry out this work was implicit in the project grant that funded it. No additional permission was required to carry out this work.

### 2.2 Statistics

All analyses were performed using R software (version 4.4.0, R Core Team 2024). A complete list of the packages used can be found in Supporting Information S1.

We modeled variation in egg volume as a function of multiple covariates of interest. Because using a standard equation derived for great tits might conflate possible changes in different egg dimensions responsible for modifications in egg volume, we investigated three additional morphological measures: egg width, length, and sphericity in supplementary analyses. Since clutch identity accounted for most of the variation in egg volume, we further explored, in a supplementary analysis, predictors of differences between clutches in mean egg volume. We used hierarchical generalized additive mixed models (GAMMs) implemented in the *mgcv* R package (Wood 2017; Pedersen et al. 2019). This flexible modeling framework allowed us to account for non‐linearity in preliminary graphic assessments of the relationships between variables. Additionally, GAMMs can incorporate non‐linear temporal autocorrelation when time is included as a covariate (Simpson 2018), which is relevant in the context of sequential egg‐laying. We modeled the data using a Gaussian distribution with an identity link function. The models were fitted using fast restricted maximum likelihood (fREML), as this method has been shown to provide more robust estimates of the optimal penalization for the wiggliness of smooth functions compared to other available approaches (Wood 2017). To ensure model adequacy, we evaluated the residual distributions and patterns (Figures S4–S9), the sufficiency of the number of basis dimensions (Tables S1–S5), checked for concurvity (i.e., generalized form of collinearity) between smooth functions (Tables S6–S11), and assessed the contribution of each variable to deviance (Table S1) and variance explained (Tables S17–S21) using standard GAM diagnostic procedures. Significance tests for smooth functions were carried out using a Wald‐like test of the null hypothesis that the smooth function is a flat horizontal function implemented in the mgcv package (see Wood 2017, section 6.12, Tables S22–S25). Because variation between clutches is secondary to the main interest of this article, we provide a further description of these analyses and their results in the Supplementary Information S1. The data and R code used in these analyses are available at https://doi.org/10.5281/zenodo.15161123.

### 2.3 Within Clutches: Egg Model

The response variable, egg volume, was standardized (scaled hereafter) based on the entire dataset (*N*_egg_ = 1084). Transformation and modeling of egg width, length, and sphericity were equivalent to those carried out for egg volume and can be found in Supporting Information S1 (clutch models).

### 2.4 Main Effects

Smooth functions of three covariates were included using cubic regression splines: First, egg position in the laying sequence was incorporated to assess potential differences in volume investment, as predicted in the “brood survival” and “brood reduction” hypotheses (Slagsvold et al. 1984), maturation of ovary, or carryover costs of egg production (Parsons 1976). Second, the clutch size was included as a proxy for overall reproductive investment, which is expected to be negatively correlated with egg volume (Blackburn 1991; Christians 2000; Williams 2001; Song et al. 2020). Third, the mean minimum temperature during the 3 days of egg formation was considered, given that lower temperatures may increase energetic demands for thermoregulation, potentially reducing egg volume (Järvinen and Ylimaunu 1986).

The egg position in the laying sequence was scaled within each clutch using standard scoring, while the other two independent variables were scaled across the entire dataset. Pearson correlation coefficients were used to assess collinearity among variables, and highly correlated variables were excluded to prevent concurvity in the model (Figure S3). As a result, the egg laying date was not included in the models, despite its potential effect on investment strategies, due to its strong negative correlation with minimum temperature (see Supporting Information S1).

### 2.5 Interactions

Additionally, we included two interactions. The interactions between egg‐laying order and clutch size and minimum temperature and clutch size were modeled as isotropic bivariate smooths using thin plate splines. Isotropic smooths were chosen because all interacting variables were scaled, meaning they shared the same unit of measurement (standard deviations) (Wood 2017). First, the interaction between egg laying order and the clutch size was included, as investment strategies have been observed to vary depending on clutch size (Christensen and Balsby 2020). Second, an interaction between minimum temperature and clutch size was incorporated to account for differences in competitive pressure during egg formation. Since each egg develops in the oviduct for 3 days, a given position in the laying order does not imply the same competition in all clutches. For example, the fourth egg develops alongside four other eggs in a clutch of six but only two in a clutch of four. This difference in competition could influence how resources are allocated, and temperature effects may differ depending on the overall intensity of competition within the clutch, justifying the inclusion of this interaction.

### 2.6 Random Intercepts and Slopes

We included two random intercepts: year and clutch identity. The inclusion of the year was justified by visual inspection, which revealed variation in clutch size and mean egg volume across years, suggesting potential dependency driven by fluctuations in resource availability due to changes in rainfall or other abiotic and biotic factors (see Figure S1). Since egg volume was scaled using the entire dataset, including clutch identity as a random intercept allowed us to estimate the variation attributable to female identity, which serves as a proxy given that individual identification was unavailable for the early years of this long‐term study. Additionally, accounting for clutch identity helped control for the dependence inherent in data collected within the same clutch.

We also considered incorporating a factor smooth interaction (analogous to a random slope) to model the effect of egg position in the laying sequence for each clutch. This approach aimed to capture potential variation in laying strategies among females, as suggested by preliminary data visualizations. However, including this random slope led to non‐normality in residuals and high concurvity (Figure S5, Table S7). Given that the full model, without this term, did not exhibit such violations (Figure S4, Table S6), we opted for its exclusion in the final analysis (see Section 3).

### 2.7 Temporal Autocorrelation

To account for the dependence in the egg‐laying process, understood as a time series, we incorporated an autocorrelation structure in the model residuals. Since the *mgcv* package requires specifying the rank of the first‐order autocorrelation process, we first ran the full egg model both with and without random slopes, assessing the presence of autocorrelation in their residuals. To do this, we correlated the residuals of each model with a lagged version, excluding matches from different clutches. In both cases, we detected moderate but significant autocorrelation, albeit with opposite signs (full model without random slopes: Pearson coefficient = 0.19, *t* = 5.92, df = 932, *p* < 0.001; full model with random slopes: Pearson coefficient = −0.24, *t* = −7.57, df = 932, *p* < 0.001). We re‐ran both models, this time incorporating the autocorrelation structure corresponding to each case. For partial models analyzing the contribution of each variable, we used the autocorrelation level of the full model without random slopes, as these models did not include the random slopes.

### 2.8 Model Diagnostics

The model appeared correctly specified regarding *k*‐basis dimensions, considering the reduction in degrees of freedom after penalization and the *k*‐indexes (Table S1). No violations of normality of residuals or homoskedasticity were detected. Observed concurvity was generally low and arose mainly from random factors. It exceeded 50% in only two cases (Table S6). The first involved the random intercept of clutch identity and minimum temperature (concurvity = 0.82), indicating that different clutches were exposed to distinct temperature ranges as the season progressed. The second involved the smooth function of clutch size and its interaction with minimum temperature (concurvity = 51%). Overall, the GAMM model met the necessary assumptions for investigating the effects of explanatory variables.

### 2.9 Dependence Between Investments

As a further step in the analysis, we examined whether the raw residuals from the full egg GAMM exhibited any systematic pattern. Specifically, we tested whether, beyond the strategy employed by each female regarding egg volume as a function of laying order, investments in individual eggs were influenced by investments in other eggs within the same clutch. This two‐step approach can be understood as follows: while the previous model captured the non‐linear trend in investment over the laying sequence within each clutch, this step evaluated whether deviations from that trend exhibit internal dependencies. To investigate this, we computed both autocorrelation (ACF) and partial autocorrelation (PACF) of the residuals for each clutch using the acf() and pacf() functions in R. The acf() function estimated the correlation between each value in a series and its lagged values. The pacf() function, in turn, fits an autoregressive model, where each value in the series is modeled as a function of its past values, with coefficients estimated by minimizing squared errors (Shumway and Stoffer 2017). To determine statistical significance of the observed values, we calculated the non‐significant interval for a white‐noise series using the equation $\pm \frac{2}{\sqrt{\bar{n}}}$, where $\bar{n}$ represents the mean clutch size. Given that these methods are typically applied to large time‐series datasets, whereas our dataset consists of multiple short series, we also computed the mean ± standard deviation of the autocorrelation coefficients for each lag. Finally, we assessed the significance of the results by examining whether this interval overlapped with zero.

## 3 Results

**FIGURE 1:**
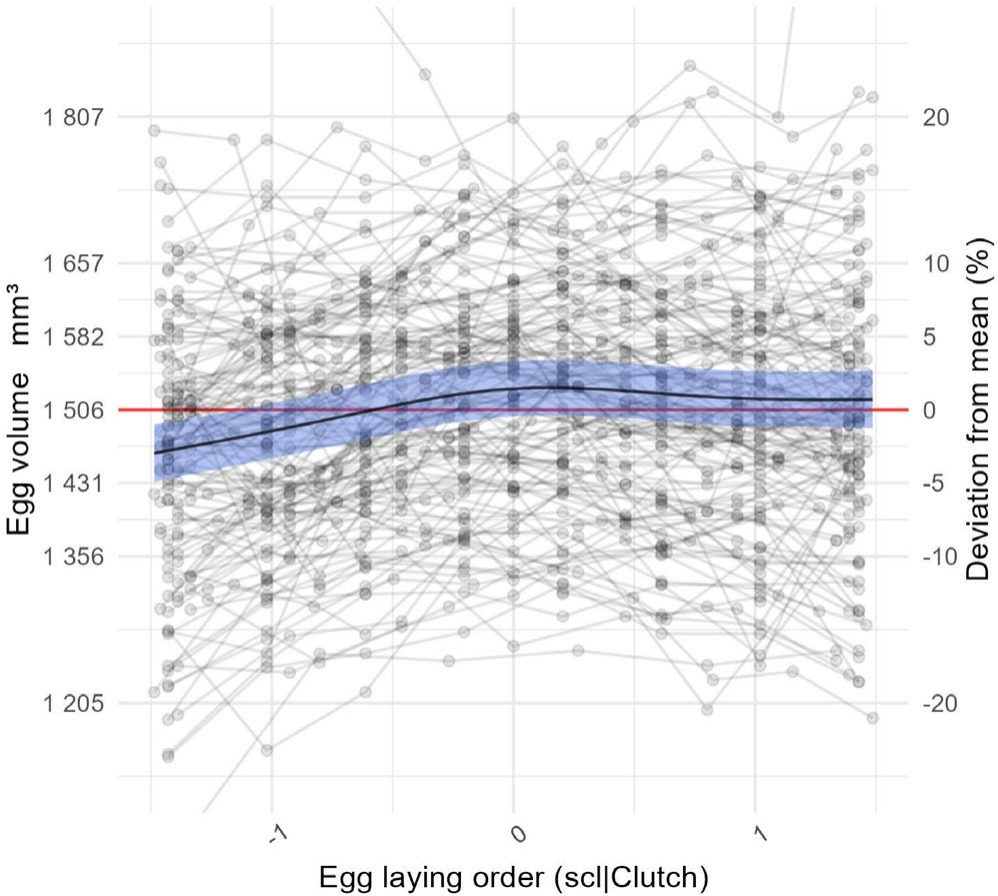
Egg volume as a function of laying order: The *x* axis shows the position in the egg laying order of a given egg scaled per clutch size, *y* axis on the left shows the egg volume in mm^3^ while the *y* axis on the right represents egg volumes as deviation in percentage from the mean egg volume. The points show values observed and gray lines connect values from eggs in the same brood. The red line indicates the mean egg volume observed. The black line indicates the predicted values by the GAMM model excluding effects of other variables with the 95% confidence interval in shaded blue.

**FIGURE 2:**
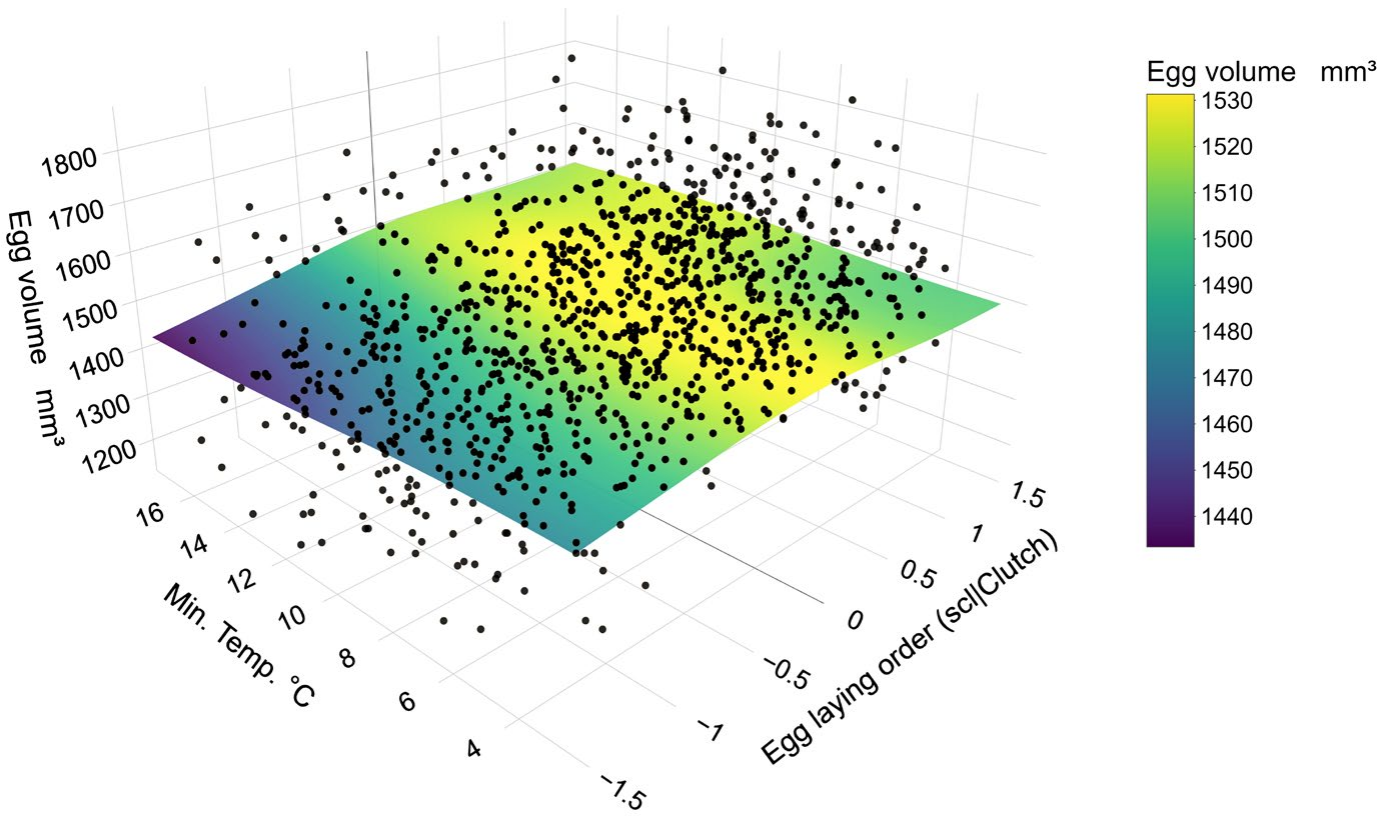
Egg volume as a function of laying order and minimum temperature: The *x* axis shows the position in the egg laying order of a given egg scaled per brood size, *y* axis shows the temperature in °C and the *z* axis on the left shows the egg volume in mm^3^. The black points show values observed. The 3D surface depicts the predicted values based on the egg full model without random slopes. Bluer colors indicate smaller and yellower bigger eggs.

The egg full model explained 81.1% of the deviance observed in the null model. Females differed in their mean egg volume (mean = 1506.24 mm^3^, coefficient of variation = 7.69%), and as a consequence, the random intercept per clutch accounted for the largest portion of deviance explained, 78.2% (*F* = 19.2, *p* < 0.001; Tables S12 and S22, Figure S10). All terms including egg position, as a main effect and as an interaction, contributed 2.88% (main effect *F* = 3.83, *p* < 0.001; Figure 1, Tables S12 and S22, Figure S15), while the interaction between minimum temperature and egg position in the laying order contributed 0.37% (*F* = 17.26, *p* = 0.076; Figure 2, Tables S12 and S22). Other variables did not significantly contribute to the explanatory power of the model (Table S12, Figures S11–S15). Additionally, the calculation of variance components supported these findings regarding the partial effects of each variable (Table S17, Variance).

After accounting for inter‐female variation, first‐laid eggs tended to be slightly smaller than the clutch mean, followed by a gradual increase in size, peaking towards the middle of the laying sequence and then decreasing again, with the last eggs returning to the clutch mean (see Figure 1). This laying order effect was relatively small, with deviations ranging between circa −2.5% and +2% from mean egg volume. Additionally, supplementary analyses indicate that the non‐linear increase in egg volume as a function of laying order appears to be mediated by a saturating increase in egg width (−2% to +0.75% of width mean; see Figure S20, Table S23) concurrent with a less pronounced two‐step decrease in egg length (+0.5% to −0.5% of length mean; see Figure S21, Table S24). These changes in egg width and length resulted in a non‐linear increase in egg sphericity as laying order progressed (−2.5% to +1.75% of sphericity mean; see Figure S22, Table S25). Thus, eggs became wider, shorter, more spherical, and more voluminous as the laying order progressed.

Minimum ambient temperature during the formation did not significantly affect egg volume (Table S22, Figure S13). However, the interaction between egg position and temperature showed a non‐significant trend, suggesting that temperature changes could influence egg volume differently depending on laying order (*F* = 17.26, *p* = 0.076; Figure 2, Table S22), albeit limited effect size, circa −2.5% and +2% from mean egg volume. While middle eggs remained relatively stable, first‐laid eggs were smaller at higher temperatures. In contrast, later‐laid eggs were smaller at lower temperatures, though the magnitude of this effect was weaker than the observed for first‐laid eggs.

Clutch size did not significantly affect egg volume either as a main effect or through interactions (Figures S12 and S15, Table S22). There was a non‐significant trend of limited effect size indicating that larger clutches contained slightly larger eggs (circa −2.5% and +2% mean volume), with the notable exception of a single four‐egg clutch, which had a mean egg volume higher than all others (Figure S12). Interannual differences in egg volume were limited and not significant (Figure S11, Table S22).

**FIGURE 3:**
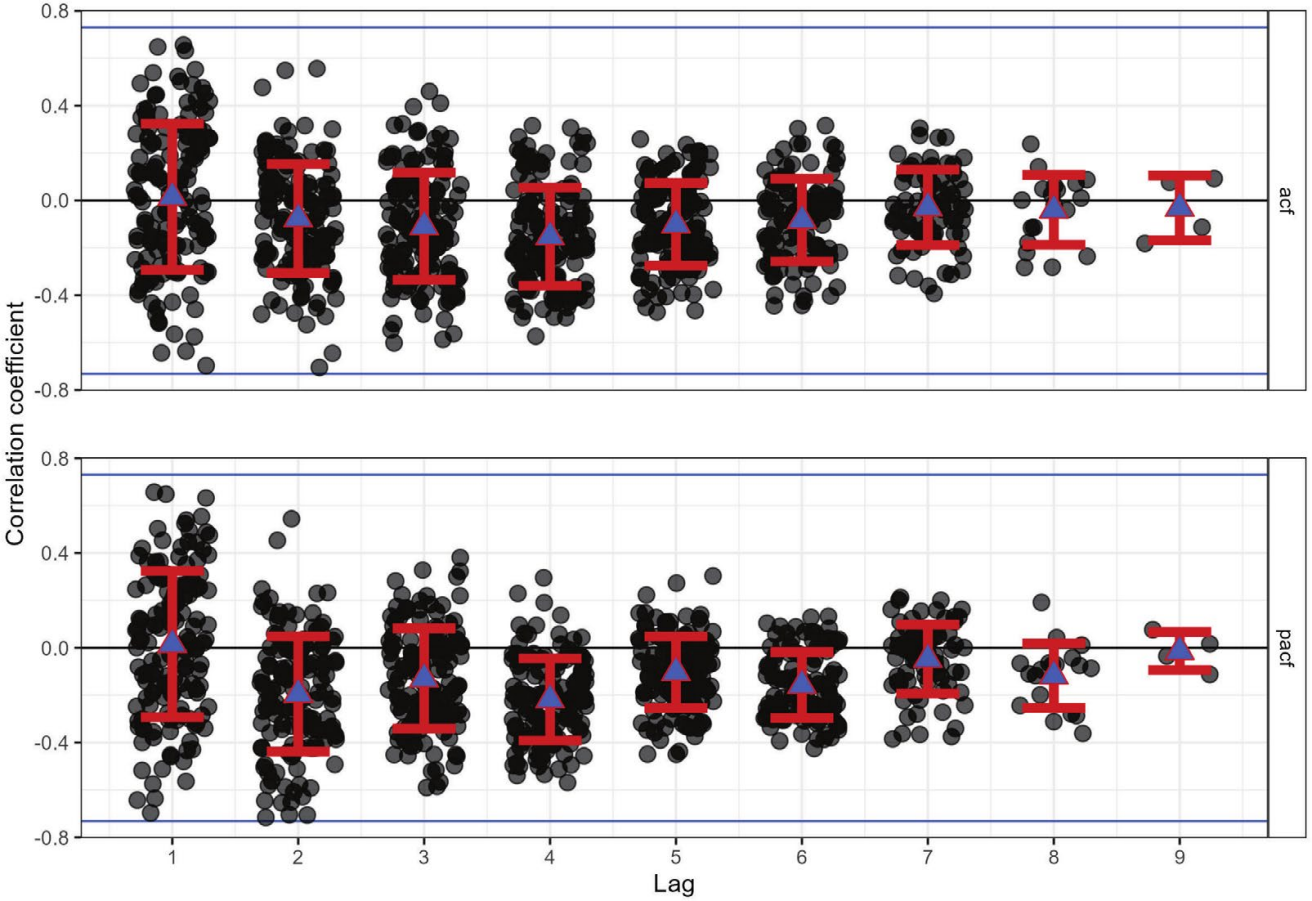
Autocorrelation coefficients for standardized residuals in the egg full model without random slopes: The *x* axis represents the lag in days within a clutch between two residuals of egg volume. The *y*‐axes indicate the Pearson correlation coefficient or the coefficient from autoregressive models. Points represent calculated values for individual clutches, while blue triangles show the mean correlation for each lag with standard deviations represented by error bars. The upper panel displays autocorrelation values, and the lower panel shows partial autocorrelation functions. Black lines mark a correlation of 0, while blue lines delineate the non‐significant range, which accounts for expected values under a white noise series with a sample size equivalent to the mean clutch size.

The standardized residuals of the models did not exhibit significant within‐clutch correlations when compared to a white noise series of similar size (Figure 3). All mean correlations per lag between two observations fell within the non‐significant range. However, when analyzing lags within and between raw and standardized residuals, certain patterns emerged. The autocorrelation structure implemented in the model effectively reduced the mean correlation for lag one to zero (Figure 3, Figure S16). Moreover, while autocorrelation between raw residuals of eggs laid 1 day apart was generally positive (Figure S16), the correlation between eggs further apart in the laying sequence tended to shift towards negative values. Thus, investments beyond or below the predicted strategy seemed to occur in pairs of eggs and might have entailed competition with eggs coming later in the laying order.

## 4 Discussion

Changes in egg volume within clutches have been explained either as an adaptive manipulation of offspring phenotypes or as a by‐product of females' physiological regulation. We found evidence for consistent patterns in egg volume variation according to laying order in this great tit population, albeit of small magnitude (approximately 5% of the mean egg volume). Our results show that the first eggs in the clutch were slightly smaller than average, whereas middle and later eggs tended to be slightly larger. Neither the minimum temperatures during egg formation nor clutch size significantly predicted egg volume or differences in mean egg volume between clutches (Table S23, Figures S17–S19), although some trends were observed. Finally, most of the variation in egg volume was explained by clutch identity. We discuss the adaptive, by‐product, and methodological implications of our results in the next sections.

### 4.1 Variation in Egg Volume as a Tool for Strategic Offspring Prioritization?

In a meta‐analysis, Krist (2011) shows that, across bird species, the egg size is strongly correlated with fitness proxies such as hatchling mass and condition (circa 0.8 and 0.6, respectively; see figure 4 in Krist 2011). Moreover, egg size is consistently associated with later body mass (circa 0.3 from nestling to post‐fledging stages) and survival (circa 0.2 for both nestling and post‐fledging). According to the adaptive manipulation set of hypotheses regarding within‐clutch variation in investment, some species aim to enhance the survival of the entire brood (the “brood survival” hypothesis), while others prioritize larger chicks at the expense of smaller ones (the “brood reduction” hypothesis; Slagsvold et al. 1984). Thus, variation in egg volume appears to be a key maternal investment that can be adaptively adjusted throughout the laying sequence.

In this study, we found that egg volume tends to increase as laying progresses. However, we also observed a slight decrease in volume between the middle and last eggs, suggesting that females prioritize the middle eggs, invest secondarily in the last ones, and allocate the least investment to the first. In approximately 63% of clutches in the study population, females begin incubation after completing the clutch (Álvarez and Barba 2014). Combined with the increasing egg volume pattern observed here, this suggests that in synchronously incubated clutches, females may be favoring middle and late eggs over the first‐laid ones. In the remaining 37% of clutches, incubation begins before clutch completion, typically with 76% of these cases starting when the penultimate egg is laid (Álvarez and Barba 2014). In such asynchronous clutches, middle eggs still enjoy an advantage over the first ones, being larger and hatching at the same time. Meanwhile, although the last egg hatches later, its larger size might compensate for the delay, potentially placing it on a more equal footing with earlier hatchlings. If this pattern holds, females may be employing a mild form of brood reduction—not in the conventional sense of disadvantaging the last eggs but rather by disadvantaging the first.

In blue tits (*Parus caeruleus*), Nilsson and Svensson (1993) found that the first egg was generally smaller and that in asynchronously incubated nests, the last egg was significantly larger compared to synchronously incubated ones. Thus, the shape of the egg volume investment pattern observed in the present study might have been influenced by the females' incubation strategies. Further research measuring incubation behavior alongside egg size throughout the laying period in great tits is needed.

Although the observed changes in egg volume are relatively small (circa 5%), our results suggest that, overall, females follow a brood survival strategy while slightly favoring middle eggs—though not to the extent that clearly supports a brood reduction scenario. Additionally, due to the modest effect size of laying order on egg volume, the significance of this variation for long‐term fitness outcomes remains uncertain. While survival and body condition correlate with egg volume, implying potential effects on lifetime reproductive success, interpretations should be made cautiously. First, Krist (2011) used Pearson's correlation coefficient as an effect size measure, which reflects the signal‐to‐noise ratio rather than the magnitude of biological impact. Second, studies in tits suggest that although egg size influences hatchling mass and early growth, these differences often diminish as nestlings age (Schifferli 1973; Nilsson and Svensson 1993; You et al. 2009). Further studies on the fitness consequences of egg volume and laying order in great tits and other species are necessary to determine the biological relevance of these findings.

In altricial species, parents can further adjust post‐hatching care to either reinforce or compensate for initial differences among chicks, affecting survival and reproductive potential. It is, therefore, important to consider post‐hatching parental investment when interpreting reproductive strategies related to within‐clutch resource allocation. For example, You et al. (2009) found that in a Chinese great tit population, although later‐laid eggs tended to hatch later, parents compensated by feeding them more frequently, therefore mitigating size disadvantages. Conversely, in a Korean population, Kang and Lee (2023) found no evidence of selective feeding based on laying order. More research is needed to determine whether the relationship between laying order and egg volume is shaped by both pre‐ and post‐hatching investment and to clarify which hypothesis—brood survival or brood reduction—is better supported.

### 4.2 Beyond Adaptation: Energetic Costs and Reproductive Organ Maturation?

The next set of hypotheses explains variation in egg volume as a by‐product of the female's changing capacity to invest in egg formation during the laying process. According to these hypotheses, the pattern observed in our results—an initial increase in egg volume followed by a slight decrease—does not necessarily imply that females prioritize middle offspring. Instead, it may result from a combination of reproductive organ maturation and the energetic costs associated with egg production (Parsons 1976; Järvinen and Ylimaunu 1986; Wiebe and Bortolotti 1996). As reproductive organs mature, egg volume is expected to increase over the course of laying. However, if females progressively deplete their energetic reserves, the volume of later eggs may be reduced. Thus, the observed pattern could reflect these two processes—maturation and increasing energetic costs—acting simultaneously throughout the laying sequence.

Although we lack direct data on reproductive organ maturation, we observed consistent increases in egg volume with laying order across clutches throughout the season (Figure S2). This observation suggests that even “late” clutches (see Section 2.1), for which reproductive maturation of reproductive organs should already be complete at the onset of laying, exhibited the same volume pattern. Therefore, it is unlikely that the observed effects of laying order are driven by seasonal reproductive maturation. Nonetheless, the concurrent changes we observed in egg width and length may indicate short‐term maturation processes operating within individual clutches, potentially enabling the production of wider, shorter, and more spheric eggs as laying progresses (Supporting Information S1 within‐clutch models, Figures S20–S22).

Our results provide mixed evidence regarding the role of energetic trade‐offs in shaping egg volume. First, middle eggs often develop concurrently with a greater number of other eggs, particularly in larger clutches. Despite this, middle eggs tended to be the largest, and we found no effect of clutch size on this pattern. Supporting the idea that energetic trade‐offs during egg formation may not be the primary driver, we found no significant effect of minimum ambient temperatures, which would be expected to reflect competing demands between thermoregulation and egg production. Interestingly, we did find a trend for larger first eggs under lower temperatures, suggesting that females may compensate for more challenging conditions by investing more in early eggs. Since temperature did not predict mean clutch volume (see Supporting Information S1 between‐clutch model, Figure S19), this pattern may reflect an effort to reduce the disadvantage of first‐laid eggs, further supporting a brood survival strategy.

Given that our study population is located in the Mediterranean region, ambient temperatures experienced during laying are generally warmer than in the populations examined in earlier studies (e.g., Järvinen and Ylimaunu 1986). These authors found that, in the Pied Flycatcher, the deviation of the last egg from the mean egg volume (*D*) was negative when temperatures were below 6°C and positive at higher temperatures, with greater differences under higher temperature variability. If we had used *D*, our results would align with their findings: later eggs tended to be larger, and first eggs had lower mean volumes in warmer conditions. While the *D* metric supports the idea of changes driven by energetic constraints, the investment in first eggs under cold conditions suggests that this explanation is incomplete.

At the same time, we found some indication that energetic trade‐offs among eggs may influence volume allocation. Residuals from the full model suggest that when females invest heavily in a specific egg, the volume of subsequent eggs—except for the one laid immediately after—tends to be reduced. A similar trade‐off has been described in blue tits, where females produced smaller first eggs unless supplemented with food (Nilsson and Svensson 1993), Considering the evidence from both adaptive manipulation and by‐product hypotheses, we suggest that female great tits in eastern Spain adopt a brood survival strategy while slightly favoring middle eggs, particularly under more favorable environmental conditions.

To further disentangle the mechanisms behind the observed pattern, future studies should experimentally manipulate resource availability during laying. Such approaches could clarify whether egg volume allocation arises from strategic investments under energetic constraints or from physiological limitations imposed by reproductive organ maturation at the start of the clutch.

### 4.3 Limits of Traditional Metrics and the Promise of New Analytical Tools

Interpreting our results in the context of previous studies on *Parus major* is challenging due to differences in metrics, sample sizes, and statistical approaches (Table 1). The only study that allows for a clear point of comparison—due to its focus on egg volume and a comparable sample size—is that of You et al. (2009). Their clutch size range (6–14 eggs) was broader than ours (4–10 eggs), and they modeled the relationship linearly. However, their results also suggest a non‐linear pattern within the overall increase detected. In their Figure 2, egg volume appears to increase and then slightly decline, with a slightly larger magnitude than in our population (5%–10% vs. 5% of mean egg volume). Given that other studies had much smaller sample sizes and used varying methodologies, it remains premature to draw general conclusions about the shape of the relationship between laying order and egg volume in *Parus major* . Nevertheless, non‐linear trends involving the first and middle eggs appear to be present across different populations whenever the available data permitted such assessment.

When comparing with other species (Table 1), the pattern observed in our population resembles that of blue tits, contrasts with that of pied flycatchers, and is the reverse of that reported in more distantly related species such as common eiders, budgerigars, and starlings (Table 1). In terms of magnitude, our variation is twice that reported for starlings, half that of eiders, and less than one‐third that found in pied flycatchers (Table 1). Previous studies have addressed non‐linearity either by incorporating polynomial terms or by treating laying order as a categorical variable. Our study demonstrated that GAMMs provide a flexible and robust analytical framework to account for non‐linearity and temporal autocorrelation in egg‐laying sequences.

One of the most influential studies on female reproductive strategies in relation to laying order relied on the metric *D*, the deviation of the last egg from the mean egg volume in a clutch (Slagsvold et al. 1984). We caution against the continued use of *D* in future studies based on our results and existing evidence. The choice of *D* by Slagsvold et al. (1984) was based on preliminary analyses of a single species, the Hooded Crow ( *Corvus cornix* ), where *D* was found to be highly correlated with other within‐clutch variation measures. However, if our analysis had relied solely on *D*, we would have detected no evidence of adaptive variation in egg volume since the last egg deviates only ~1% from the mean. Moreover, the widespread presence of non‐linear patterns—especially in the early portion of the clutch—reinforces the notion that *D* cannot adequately capture the complexity of variation in egg volume across laying order. In such cases, *D* might falsely suggest a similarity between species that display a decrease in later eggs while missing critical variability in middle eggs. Possibly due to the analytical challenges posed by non‐linear and temporally structured data, broad‐scale comparative analyses on within‐clutch egg‐size variation—accounting for phylogeny, ecology, and life‐history traits—are still lacking. Promising methodological advances, such as those proposed by Clark et al. (2020) GAMMs or Rosas‐Puchuri et al. (2024) using Kernel Ridge regression, could help overcome these limitations.

Finally, while laying order accounted for only 2.88% of the deviance in egg volume, nearly 80% of the variation in egg volume, width, length, and sphericity was attributable to differences between clutches. This strong among‐clutch variation is consistent with previous avian studies (reviewed in Christians 2002). Christians (2002) concluded that although females in a population differ widely in their overall capacity to invest in reproduction, each individual tends to maintain relatively stable egg volumes, primarily adjusting investment through clutch size. We found a non‐significant linear trend suggesting that eggs were slightly larger in larger clutches—a pattern supported by a significant effect in the same population using a larger sample size (Encabo et al. 2001). These results suggest inter‐individual differences in investment within the studied population, with some females investing simultaneously in both egg size and clutch size, contrary to classic life‐history trade‐off expectations.

Since egg volume and mass are associated with offspring quality (Slagsvold et al. 1984; Williams 1994; Krist 2011), our findings further suggest that female reproductive capacity may be a key target for sexual selection, particularly in monogamous species. Although we did not explicitly account for female quality, our results, combined with previous studies, support the idea that females consistently adjust egg volume, potentially maximizing overall brood success. Future research should explore how factors such as a male phenotype and the reproductive consequences of within‐ and between‐clutch egg size variation influence female reproductive success. Expanding upon the comparative framework established by Slagsvold et al. (1984) will be essential to fully understand reproductive strategies in female birds from a whole‐brood perspective.

## Author Contributions

**Lluís Socias‐Martínez:** conceptualization (supporting), data curation (supporting), formal analysis (lead), investigation (equal), methodology (equal), software (lead), visualization (lead), writing – original draft (equal), writing – review and editing (equal). **Elena Álvarez:** data curation (equal), funding acquisition (supporting), writing – original draft (equal), writing – review and editing (equal). **Louise R. Peckre:** conceptualization (supporting), investigation (supporting), methodology (supporting), visualization (supporting), writing – original draft (supporting), writing – review and editing (equal). **Emilio Barba:** conceptualization (lead), data curation (equal), funding acquisition (lead), investigation (equal), methodology (equal), project administration (lead), writing – original draft (equal), writing – review and editing (equal).

## Conflicts of Interest

The authors declare no conflicts of interest.

## Supporting information


Data S1.

## Data Availability

The data and R scripts used in this article are available at Zenodo with accompanying metadata: https://doi.org/10.5281/zenodo.15161123.
