# Supplementary material for: Unveiling Clutch Investment Strategies in Birds: A Case Study on Great Tits Using Generalized Additive Models
Source: Ecol Evol. 2025 Jul 1;15(7):e71700. doi: 10.1002/ece3.71700 (PMC12213442; doi:10.1002/ece3.71700)
Supplement: Supplementary file 1 — Data S1. [file ECE3-15-e71700-s001.pdf]

Supplementary Information

Socias-Martínez L., Álvarez E., Peckre L. R., and Barba E. 2025

Unveiling clutch investment strategies in birds: A case study on great tits using generalized additive models

Contents

Explored features in the dataset prior to analyses 2
GAMMs 4
Candidate variables 4
Supplementary between-clutch model: Analyzing differences in mean egg volumes between clutches 5
Supplementary within-clutch models: Analyzing changes in egg width, length and sphericity 6
Models diagnostics 7
Deviance 18
Variance 20
Significance tests for smooths 22
Effects covariates - Egg volume full model 24
Main effects 26
Interactions 28
Autocorrelation 30
Effects covariates - Clutch full model 31
Effects covariates - Egg width length sphericity full models 34
R Packages 37
Bibliography 37

Explored features in the dataset prior to analyses

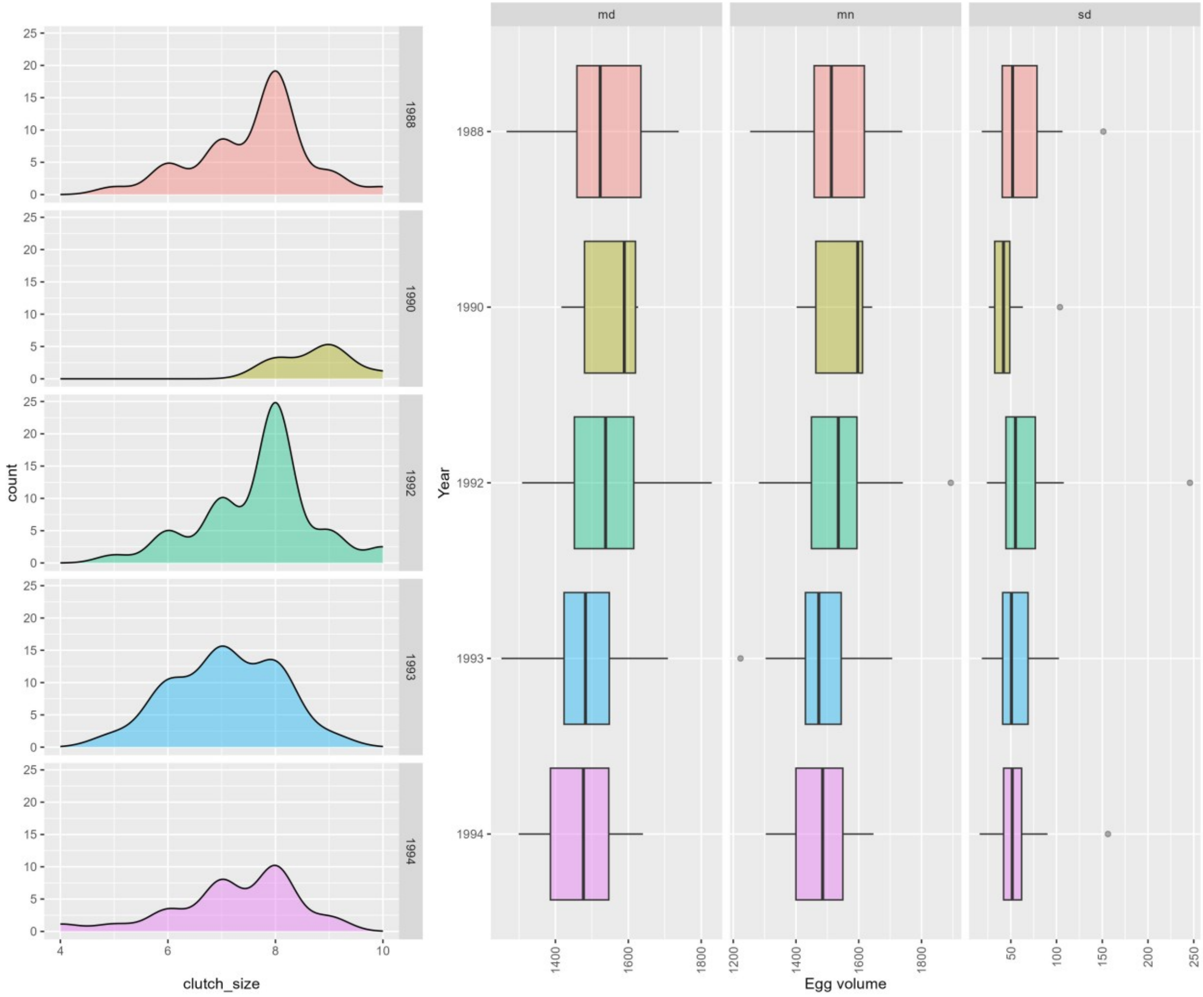

SI Figure 1 | Year to year variation in clutch and egg sizes: The left panel shows count curves for clutch sizes in different years. The x axis shows the clutch size, the y axis the number of clutches with a given size. Each row represents one of the five sampling years. In the right panel, boxplots of various measures of egg volume in different years are shown. The x axis shows egg volume, the y axis the year and each column indicated a different measure, with md the median, mn the mean and sd the standard deviation.

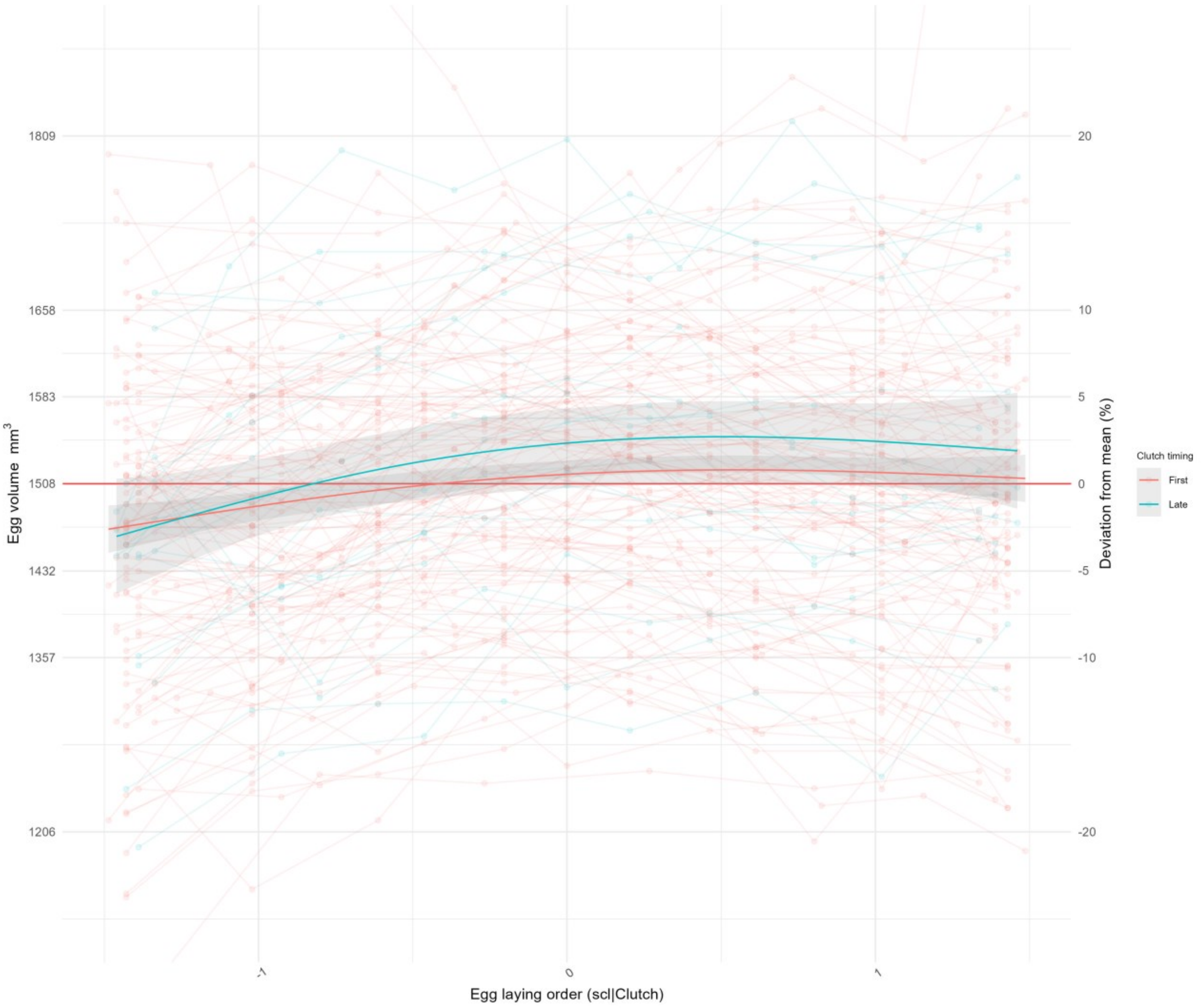

SI Figure 2 | Egg volume as a function of laying order per clutch timing: The x axis shows the position in the egg laying order of a given egg scaled per clutch size, y axis on the left shows the egg volume in mm^3 while the y axis on the right represents egg volumes as deviation in percentage from the mean egg volume. The points show values observed and lines connect values from eggs in the same clutch. The red horizontal line indicates the mean egg volume observed. The colored lines with shaded areas indicating the predicted values and 95% confidence intervals respectively generated by a preliminary GAM model fitted using package ggplot2 in R. A smooth function of laying order scaled with cubic splines was used as the only predictor of egg volume with four k basis.

GAMMs

Candidate variables

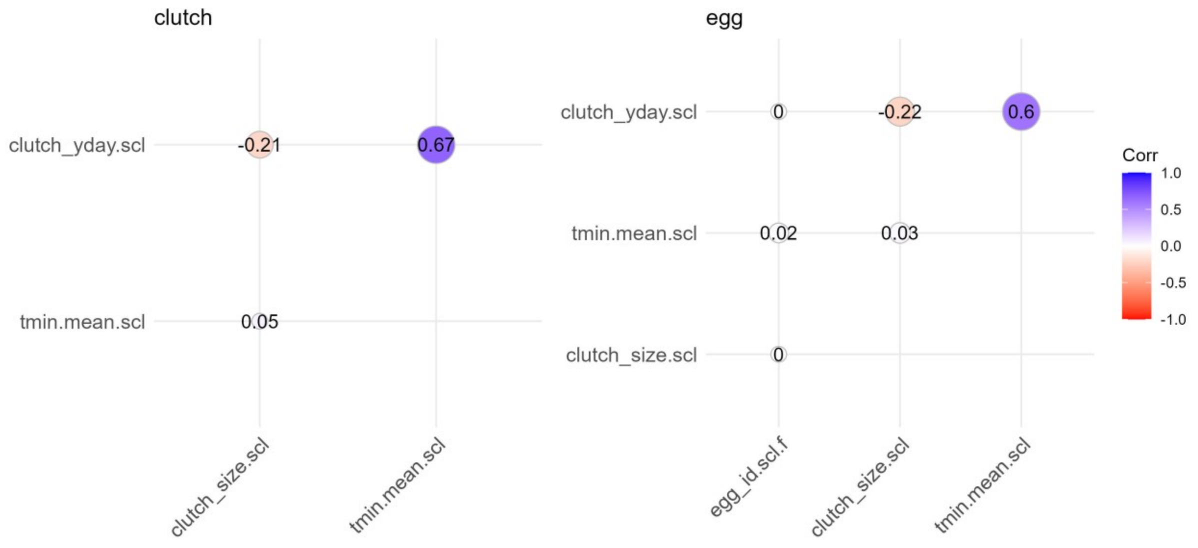

SI Figure 3 | Correlations among candidate variables: The upper correlation matrix using Pearson coefficients is depicted for the dataset used in analysis between clutches “clutch” and the within clutch level analysis between eggs “egg”. Numbers indicate the correlation coefficient, red colors indicate negative, white absence and blue positive coefficients. Variables included correspond to egg_id.scl.f”: the egg position in the laying order scaled within clutches; “clutch_yday.scl”: the day of the year the first egg in the clutch was laid, scaled; “clutch_size.scl” : the size of the clutch scaled and “tmin.mean.scl”: the mean minimum temperature during the three days of egg formation scaled.

Supplementary between-clutch model: Analyzing differences in mean egg volumes between clutches

The response variable, egg volume, was scaled prior to model fitting using the entire dataset aggregated at the clutch level (n_{clutch}=145).

Main effects

We included the minimum temperature experienced by the eggs during formation as it is suggested to induce a thermoregulation tradeoff. Specifically, we recorded the minimum temperature for the three days preceding each egg-laying date, averaged these values per egg, and then computed the overall mean for each clutch. Additionally, we included clutch size as a covariate, as life-history theory predicts that a higher number of offspring should lead to a reduced investment per individual. All values were scaled using the clutch-level dataset. As in the previous section, we assessed correlations among candidate variables to avoid issues of concurvity. Following this assessment, egg-laying date was excluded due to its strong correlation with minimum temperature (see SI).

Although the effect of temperature might depend on clutch size, warranting the inclusion of an interaction term, this was not feasible due to the limited number of observations (n_{clutch} = 145).

Random effects

A random intercept per year was included to account for year-to-year variation (see previous section).

Results

The model appeared correctly specified regarding k-basis dimensions, considering the reduction in degrees of freedom after penalization and the k-indexes (SI, Diagnostics). No violations of normality of residuals or homoskedasticity were detected. Concurvity was generally low and arose mainly from random factors. Observed concurvity never exceeded 50%. Overall, the GAMM models met the necessary assumptions for investigating the effects of explanatory f variables.

The full clutch GAMM model explained 8.68% of the deviance. The random intercept per year accounted for 7.2% of the deviance in the clutch null model, and its effect was significant (F = 1.687, p = 0.04). Removing the minimum temperature from the full model resulted in a 3.95% loss in explained deviance. However, its effect within the full model was not significant (F = 1.016, p = 0.365). In contrast, removing clutch size had a minimal impact, leading to a slight improvement in the explained deviance by 0.06%. Its effect within the full model was also non-significant (F = 0.636, p = 0.426).

Supplementary within-clutch models: Analyzing changes in egg width, length and sphericity

The response variables, egg width, length and sphericity were standard scored (scaled hereafter) based on the entire dataset (N_{egg} =1,084).

Main effects, interactions and random effects

Main effects and interactions of egg laying order, minimum temperatures and clutch size, and random intercepts of clutch identity and year were included in the same manner as for the within-clutch egg volume model.

Results

The models appeared to suffer from unmodelled structure according to the results of the kcheck() function from package mgcv (SI, Diagnostics). Egg width: clutch size shows a significant p-value and a low k-index, but the penalization having reduced the edf from five to one indicates that this might not be a problem; the interaction between egg position and minimum temperature might have suffered from a too low value of k, this is not surprising given that we have constrained them to avoid concurvity with the main effects when designing the model structure. Egg length: clutch size as per egg width; the unmodelled structure in the data from egg position is almost significant, and judging by the edf being close to k', it does indicate that a richer set of basis would have likely captured more structure. Nevertheless, we constrained k this to avoid modelling temporal autocorrelation between adjacent eggs. Egg sphericity: this model did not appear to suffer from the unmodeled structure in the data according to the kcheck() function. No violations of normality of residuals or homoskedasticity were detected. Concurvity was generally low and arose mainly from random factors or between smooths that included the same variable as a main effect and as an interaction. Observed concurvity exceeded 50% in some cases| Egg width: clutch identity and minimum temperature 54%, clutch identity and the interaction between minimum temperature and clutch size 91%; Egg length: clutch identity and minimum temperature 82%, clutch identity and the interaction between minimum temperature and clutch size 83%; Egg sphericity: clutch identity and minimum temperature 82%, clutch identity and the interaction between minimum temperature and clutch size 83%, clutch identity and the interaction between minimum temperature and egg position 61%.

The different models with other egg dimensions as responses performed similarly in the overall explained deviance and in the structure of the deviance explained by each smooth function. The majority of deviance was explained by clutch identity in all models: width 74%, length 78% and sphericity 71%. The deviance explained gathered by the main effect of egg position and its interactions differed: width 6.75%, length 1.49%, sphericity 6.40%. Other variables did not reach 1%, indicating that when the main effect of laying order has been taken into account, its interactions do not explain a significant part of the deviance.

Models diagnostics

Smooth basis dimensions

Smooth basis dimensions egg_volume fullnoRS

	k'	edf	k-index	p-value
s(clutch_id.fct)	145	133.46	NA	NA
s(year.fct)	5	1.85	NA	NA
s(egg_id.scl.f)	4	3.43	1.01	0.6
s(tmin.mean.scl)	5	1	0.98	0.3
s(clutch_size.scl)	5	1	0.83	0
s(egg_id.scl.f,clutch_size.scl)	2	0	1.01	0.67
s(tmin.mean.scl,clutch_size.scl)	2	0	1.01	0.65
s(egg_id.scl.f,tmin.mean.scl)	2	1.63	0.92	0

SI Table 1 | Evaluation of K-basis dimensions (egg volume full model): The values computed to test the sufficiency of the number of bases for each smooth are depicted. Each line in the table concerns a smooth function, the columns show the corresponding values of "k'": the implemented number of bases; "edf": the number of bases retained after penalized likelihood; "k-index": an estimate of the residual variance with values ≥ 1 indicating adequacy; "p-value": an estimate of significance computed by comparing the distribution of differences in randomly chosen residuals (the null distribution) compared to the obtained k-index. See Wood (2017), section 5.9 for more information. Variables within smooth functions concern "year.fct": the year of the nesting event; "clutch_id.fct": the identity of the clutch attempt; "egg_id.scl.f": the egg position in the laying order scaled within clutches; "clutch_size.scl" : the size of the clutch scaled and "tmin.mean.scl": the mean minimum temperature during the three days of egg formation scaled.

Smooth basis dimensions clutch_volume full

	k'	edf	k-index	p-value
s(year.fct)	5	2.63	NA	NA
s(tmin.mean.scl)	34	1.79	0.84	0.02
s(clutch_size.scl)	6	1	0.88	0.08

SI Table 2 | Evaluation of K-basis dimensions (clutch volume full model): The values computed to test the sufficiency of the number of bases for each smooth are depicted. Abbreviations and layout as per SI Table 1.

Smooth basis dimensions egg_width fullnoRS

	k'	edf	k-index	p-value
s(clutch_id.fct)	145	132.57	NA	NA
s(year.fct)	5	2.08	NA	NA
s(egg_id.scl.f)	4	3.4	1.04	0.91
s(tmin.mean.scl)	5	3.52	0.98	0.24
s(clutch_size.scl)	5	1	0.84	0
s(egg_id.scl.f,clutch_size.scl)	2	0	1.04	0.9
s(tmin.mean.scl,clutch_size.scl)	2	0.1	1.01	0.62
s(egg_id.scl.f,tmin.mean.scl)	2	1.1	0.94	0.02

SI Table 3 | Evaluation of K-basis dimensions (egg width full model): As per SI Table 1.

Smooth basis dimensions egg_length fullnoRS

	k'	edf	k-index	p-value
s(clutch_id.fct)	145	136.15	NA	NA
s(year.fct)	5	0	NA	NA
s(egg_id.scl.f)	4	3.6	0.95	0.07
s(tmin.mean.scl)	5	1	1.04	0.88
s(clutch_size.scl)	5	1	0.92	0
s(egg_id.scl.f,clutch_size.scl)	2	0.89	1	0.49
s(tmin.mean.scl,clutch_size.scl)	2	0	1	0.57
s(egg_id.scl.f,tmin.mean.scl)	2	1.41	0.98	0.21

SI Table 4 | Evaluation of K-basis dimensions (egg length full model): As per SI Table 1.

Smooth basis dimensions egg_sphericity fullnoRS

	k'	edf	k-index	p-value
s(clutch_id.fct)	145	135.66	NA	NA
s(year.fct)	5	0	NA	NA
s(egg_id.scl.f)	4	3.48	0.98	0.2
s(tmin.mean.scl)	5	1	1.03	0.81
s(clutch_size.scl)	5	1	0.98	0.19
s(egg_id.scl.f,clutch_size.scl)	2	1.02	1.03	0.82
s(tmin.mean.scl,clutch_size.scl)	2	0	0.97	0.16
s(egg_id.scl.f,tmin.mean.scl)	2	0	1.01	0.67

SI Table 5 | Evaluation of K-basis dimensions (egg sphericity full model): As per SI Table 1.

Residuals diagnostics

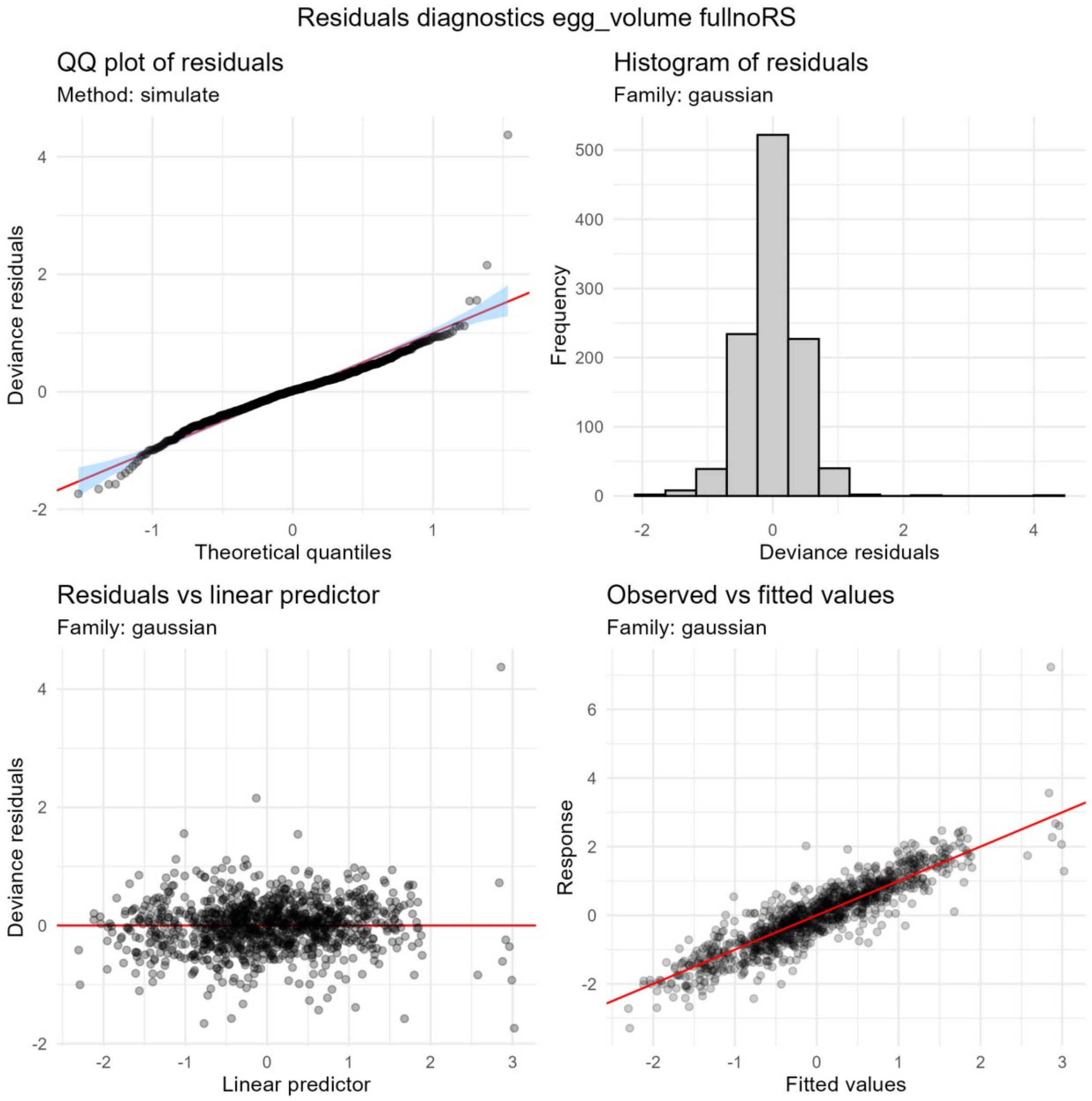

SI Figure 4 | Diagnostics of residuals (egg volume full model): A quantile-quantile plot, histogram, linear predictor-deviance residuals and fitted-response plots are depicted in the upper-left, upper-right, lower-left and lower-right panels, respectively. Transparent black dots indicate observed values and red lines reference values.

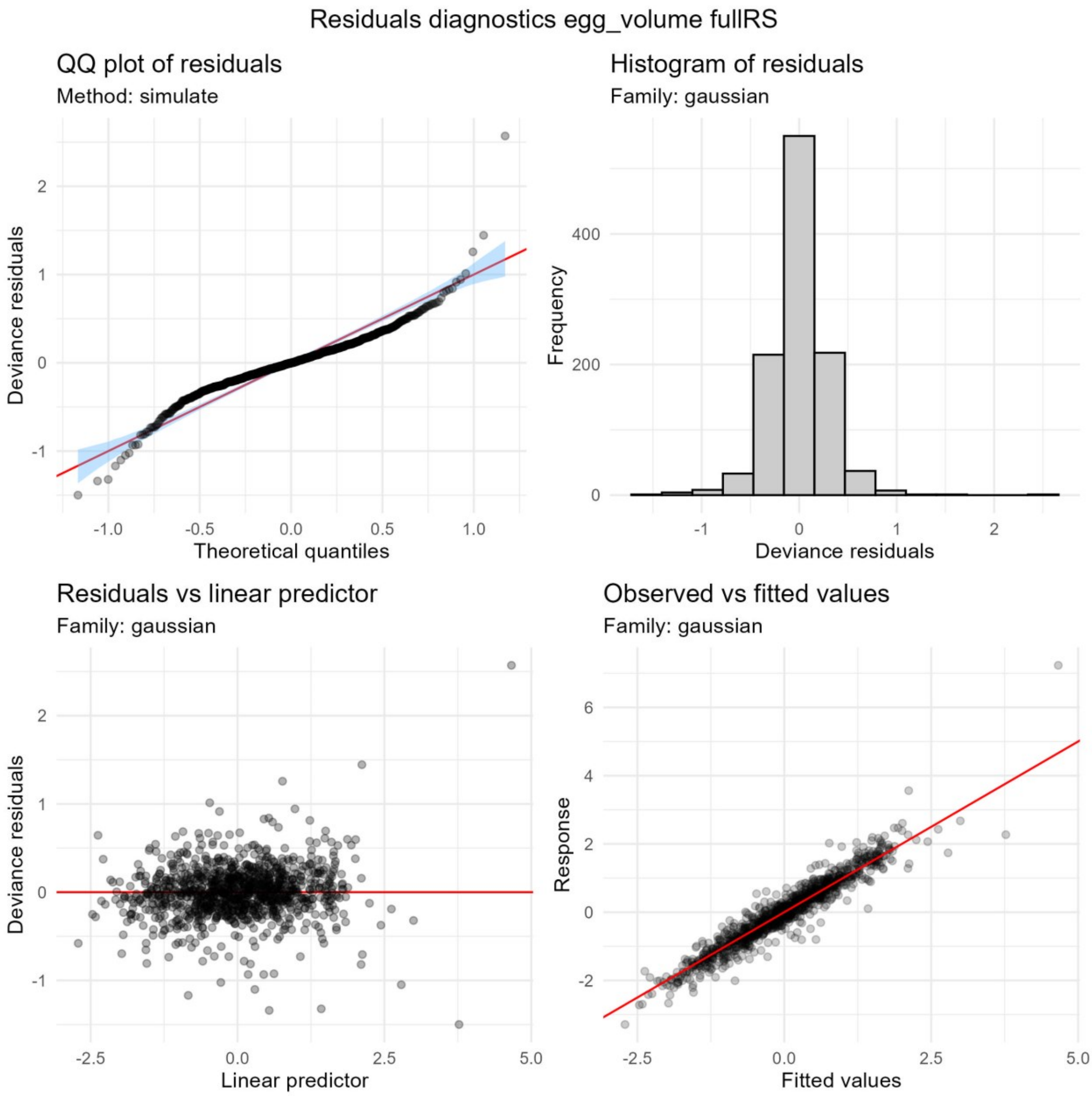

SI Figure 5 | Diagnostics of residuals (egg volume full model with random smooths): A quantile-quantile plot, histogram, linear predictor-deviance residuals and fitted-response plots are depicted in the upper-left, upper-right, lower-left and lower-right panels, respectively. Transparent black dots indicate observed values and red lines reference values.

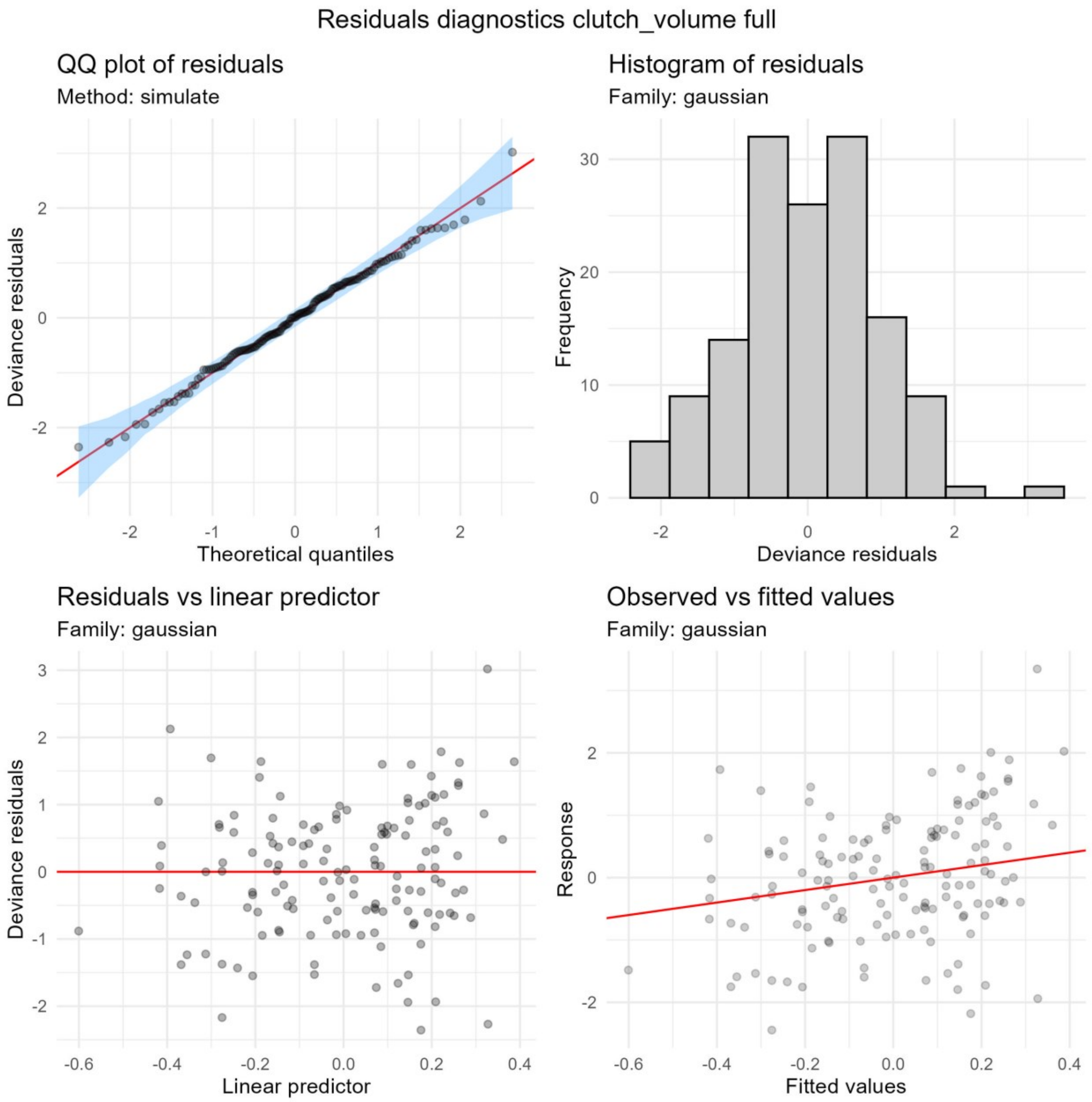

SI Figure 6 | Diagnostics of residuals (clutch volume full model): A quantile-quantile plot, histogram, linear predictor-deviance residuals and fitted-response plots are depicted in the upper-left, upper-right, lower-left and lower-right panels, respectively. Transparent black dots indicate observed values, and red lines reference values.

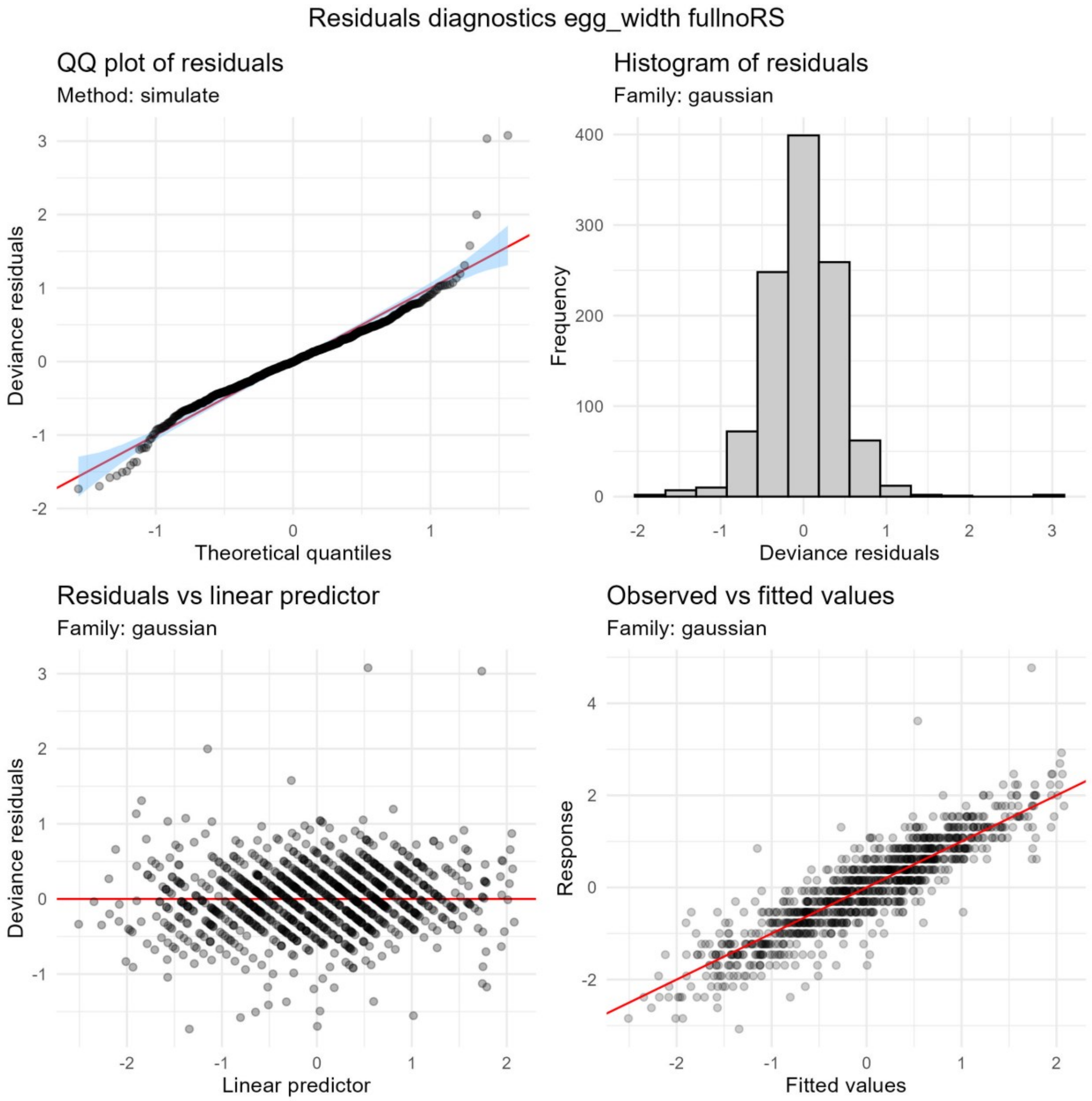

SI Figure 7 | Diagnostics of residuals (egg width full model): A quantile-quantile plot, histogram, linear predictor-deviance residuals and fitted-response plots are depicted in the upper-left, upper-right, lower-left and lower-right panels, respectively. Transparent black dots indicate observed values, and red lines reference values.

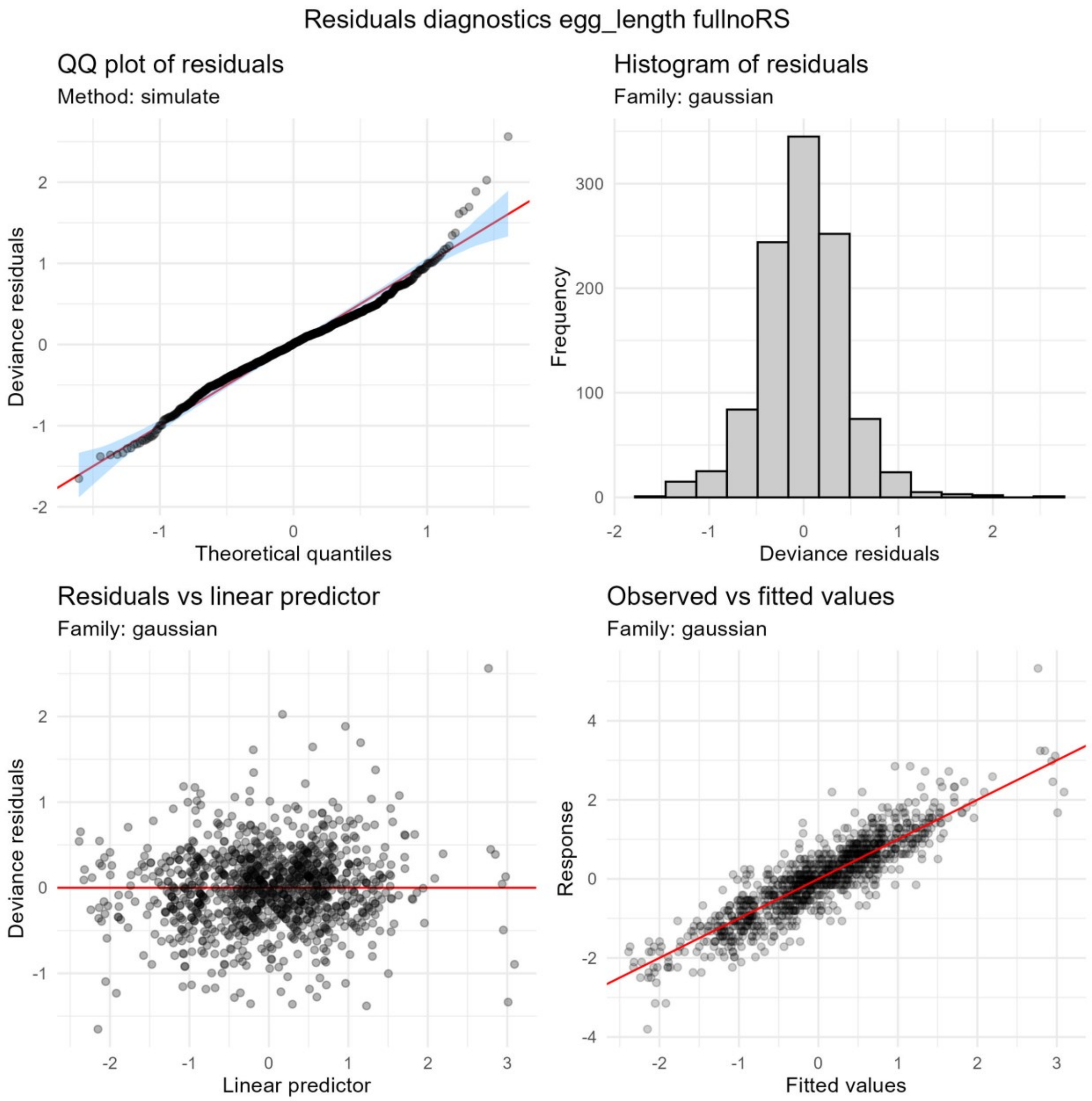

SI Figure 8 | Diagnostics of residuals (egg length full model): A quantile-quantile plot, histogram, linear predictor-deviance residuals and fitted-response plots are depicted in the upper-left, upper-right, lower-left and lower-right panels, respectively. Transparent black dots indicate observed values, and red lines reference values.

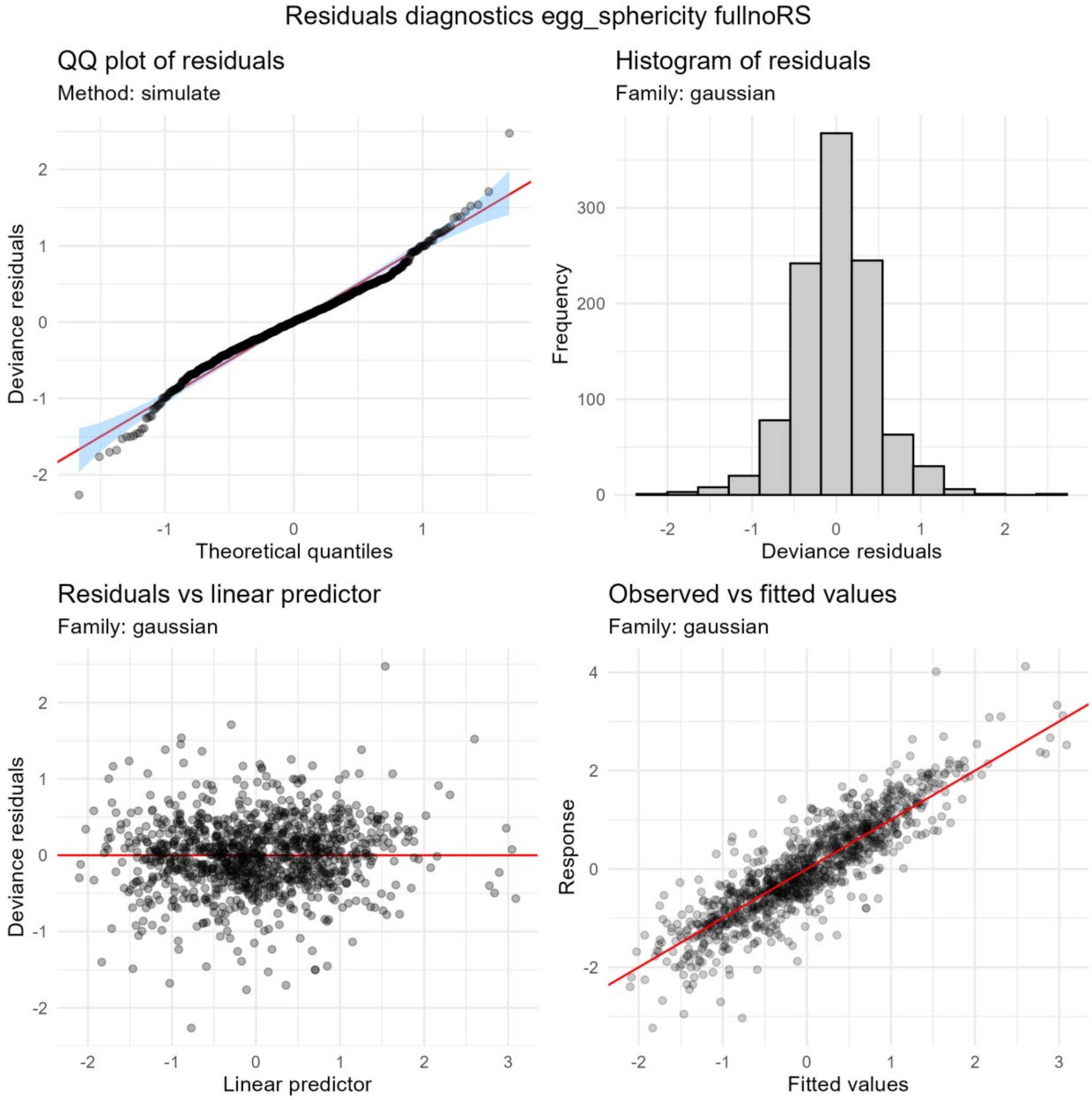

SI Figure 9 | Diagnostics of residuals (egg sphericity full model): A quantile-quantile plot, histogram, linear predictor-deviance residuals and fitted-response plots are depicted in the upper-left, upper-right, lower-left and lower-right panels, respectively. Transparent black dots indicate observed values, and red lines reference values.

Concurvity egg_volume fullnoRS

	para	s(clutch_id.fct)	s(year.fct)	s(egg_id.scl.f)	s(tmin.mean.scl)	s(clutch_size.scl)	s(egg_id.scl.f,clutch_size.scl)	s(tmin.mean.scl,clutch_size.scl)	s(egg_id.scl.f,tmin.mean.scl)
para		0	0	0	0	0	0	0	0
s(clutch_id.fct)			1	0	0.82	1	0.01	0.91	0.13
s(year.fct)				0	0.4	0.16	0	0.09	0.02
s(egg_id.scl.f)					0	0	0.05	0	0.04
s(tmin.mean.scl)						0.03	0.02	0.04	0.11
s(clutch_size.scl)							0.01	0.52	0
s(egg_id.scl.f,clutch_size.scl)								0.46	0
s(tmin.mean.scl,clutch_size.scl)									0.01
s(egg_id.scl.f,tmin.mean.scl)									

SI Table 6 | Evaluation of pairwise concurvities (egg volume full model): A matrix with the pairwise level of concurvity between smooth functions is displayed. Variables within smooth functions concern "year.fct": the year of the nesting event; "clutch_id.fct": the identity of the clutch attempt; "egg_id.scl.f": the egg position in the laying order scaled within clutches; "clutch_size.scl": the size of the clutch scaled and "tmin.mean.scl": the mean minimum temperature during the three days of egg formation scaled.

Concurvity egg_volume fullRS

	para	s(clutch_id.fct)	s(year.fct)	s(egg_id.scl.f)	s(egg_id.scl.f,clutch_id.fct)	s(tmin.mean.scl)	s(clutch_size.scl)	s(egg_id.scl.f,clutch_size.scl)	s(tmin.mean.scl,clutch_size.scl)	s(egg_id.scl.f,tmin.mean.scl)
para		0	0	0	0	0	0	0	0	0
s(clutch_id.fct)			1	0	0.23	0.82	1	0.02	0.91	0.1
s(year.fct)				0	0.01	0.4	0.16	0	0.08	0.02
s(egg_id.scl.f)					0	0	0	0.05	0	0.03
s(egg_id.scl.f,clutch_id.fct)						0.97	1	0.99	0.98	0.95
s(tmin.mean.scl)							0.03	0.02	0.09	0.07
s(clutch_size.scl)								0.01	0.63	0
s(egg_id.scl.f,clutch_size.scl)									0.57	0
s(tmin.mean.scl,clutch_size.scl)										0
s(egg_id.scl.f,tmin.mean.scl)										

SI Table 7 | Evaluation of pairwise concurvities (egg volume full model with random smooths): A matrix with the pairwise level of concurvity between smooth functions is displayed. Variables within smooth functions concern "year.fct": the year of the nesting event; "clutch_id.fct": the identity of the clutch attempt; "egg_id.scl.f": the egg position in the laying order scaled within clutches; "clutch_size.scl": the size of the clutch scaled and "tmin.mean.scl": the mean minimum temperature during the three days of egg formation scaled.

Concurvity clutch_volume full

	para	s(year.fct)	s(tmin.mean.scl)	s(clutch_size.scl)
para		0	0	0
s(year.fct)			0.48	0.15
s(tmin.mean.scl)				0.33
s(clutch_size.scl)				

SI Table 8 | Evaluation of pairwise concurvities (clutch volume full model): A matrix with the pairwise level of concurvity between smooth functions is displayed. Variables within smooth functions concern "year.fct": the year of the nesting event; "clutch_id.fct": the identity of the clutch attempt; "egg_id.scl.f": the egg position in the laying order scaled within clutches; "clutch_size.scl": the size of the clutch scaled and "tmin.mean.scl": the mean minimum temperature during the three days of egg formation scaled.

Concurvity egg_width fullnoRS

	para	s(clutch_id.fct)	s(year.fct)	s(egg_id.scl.f)	s(tmin.mean.scl)	s(clutch_size.scl)	s(egg_id.scl.f,clutch_size.scl)	s(tmin.mean.scl,clutch_size.scl)	s(egg_id.scl.f,tmin.mean.scl)
para		0	0.01	0	0	0	0	0	0
s(clutch_id.fct)			1	0	0.54	1	0	0.91	0.12
s(year.fct)				0	0.16	0.16	0	0.09	0.02
s(egg_id.scl.f)					0.01	0	0.05	0	0.03
s(tmin.mean.scl)						0.03	0.02	0.03	0.1
s(clutch_size.scl)							0	0.47	0
s(egg_id.scl.f,clutch_size.scl)								0.41	0
s(tmin.mean.scl,clutch_size.scl)									0.01
s(egg_id.scl.f,tmin.mean.scl)									

SI Table 9 | Evaluation of pairwise concurvities (egg width full model): A matrix with the pairwise level of concurvity between smooth functions is displayed. Variables within smooth functions concern "year.fct": the year of the nesting event; "clutch_id.fct": the identity of the clutch attempt; "egg_id.scl.f": the egg position in the laying order scaled within clutches; "clutch_size.scl": the size of the clutch scaled and "tmin.mean.scl": the mean minimum temperature during the three days of egg formation scaled.

Concurvity egg_length fullnoRS

	para	s(clutch_id.fct)	s(year.fct)	s(egg_id.scl.f)	s(tmin.mean.scl)	s(clutch_size.scl)	s(egg_id.scl.f,clutch_size.scl)	s(tmin.mean.scl,clutch_size.scl)	s(egg_id.scl.f,tmin.mean.scl)
para		0	0.04	0	0	0	0	0	0
s(clutch_id.fct)			1	0	0.82	1	0.05	0.83	0.09
s(year.fct)				0	0.4	0.16	0	0.05	0.01
s(egg_id.scl.f)					0	0	0.05	0	0.03
s(tmin.mean.scl)						0.03	0.02	0.19	0.06
s(clutch_size.scl)							0.05	0.04	0
s(egg_id.scl.f,clutch_size.scl)								0	0
s(tmin.mean.scl,clutch_size.scl)									0
s(egg_id.scl.f,tmin.mean.scl)									

SI Table 10 | Evaluation of pairwise concurvities (egg length full model): A matrix with the pairwise level of concurvity between smooth functions is displayed. Variables within smooth functions concern “year.fct”: the year of the nesting event; “clutch_id.fct”: the identity of the clutch attempt; “egg_id.scl.f”: the egg position in the laying order scaled within clutches; “clutch_size.scl”: the size of the clutch scaled and “tmin.mean.scl”: the mean minimum temperature during the three days of egg formation scaled.

Concurvity egg_sphericity fullnoRS

	para	s(clutch_id.fct)	s(year.fct)	s(egg_id.scl.f)	s(tmin.mean.scl)	s(clutch_size.scl)	s(egg_id.scl.f,clutch_size.scl)	s(tmin.mean.scl,clutch_size.scl)	s(egg_id.scl.f,tmin.mean.scl)
para		0	0.1	0	0	0	0	0	0
s(clutch_id.fct)			1	0	0.82	1	0.08	0.83	0.3
s(year.fct)				0	0.4	0.16	0.01	0.06	0.04
s(egg_id.scl.f)					0	0	0.05	0	0.18
s(tmin.mean.scl)						0.03	0.02	0.18	0.61
s(clutch_size.scl)							0.08	0.04	0.01
s(egg_id.scl.f,clutch_size.scl)								0.01	0.02
s(tmin.mean.scl,clutch_size.scl)									0.25
s(egg_id.scl.f,tmin.mean.scl)									

SI Table 11 | Evaluation of pairwise concurvities (egg sphericity full model): A matrix with the pairwise level of concurvity between smooth functions is displayed. Variables within smooth functions concern “year.fct”: the year of the nesting event; “clutch_id.fct”: the identity of the clutch attempt; “egg_id.scl.f”: the egg position in the laying order scaled within clutches; “clutch_size.scl”: the size of the clutch scaled and “tmin.mean.scl”: the mean minimum temperature during the three days of egg formation scaled.

Deviance

Deviance egg_volume

	model_type	null	ref	bigger	removed	null_deviance	ref_deviance
1	egg_volume	null	null	clutch_id.fct	clutch_id.fct	78.21	78.21
2	egg_volume	null	clutch_id.fct	fullnoRS	fullRS	-10.26	-47.09
3	egg_volume	null	clutch_id.fct	fullnoRS	year.fct	0.00	0.01
4	egg_volume	null	clutch_id.fct	fullnoRS	egg_id.scl.f	2.88	13.21
5	egg_volume	null	clutch_id.fct	fullnoRS	tmin.mean.scl	0.38	1.76
6	egg_volume	null	clutch_id.fct	fullnoRS	clutch_size.scl	0.00	0.00
7	egg_volume	null	clutch_id.fct	fullnoRS	egg_id.scl.f,clutch_size.scl	-0.00	-0.00
8	egg_volume	null	clutch_id.fct	fullnoRS	tmin.mean.scl,clutch_size.scl	0.00	0.00
9	egg_volume	null	clutch_id.fct	fullnoRS	egg_id.scl.f,tmin.mean.scl	0.37	1.70

SI Table 12 | Evaluation of deviance explained per variable (egg volume models): The differences in the percentage of deviance explained by a model and a reduced version of it in relation to a reference level are presented. When the consequences of removing a main effect were evaluated, its interactions were also removed to avoid the model using interactions to explain deviance. Columns gather the types of models with rows indicating the specific contrast. Column "ref" indicates the reference model from which the percentage of deviance is calculated, "bigger" the more complex model, "diff.bigger" the variable of difference between the more complex and the simpler model and "null_deviance" gathers the change in deviance explained as a percentage from deviance in the null model and "ref_deviance" in the reference model. Positive values indicate an increase in deviance explained by including a variable, and negative values indicate a decrease. Variables concern "year.fct": the year of the nesting event; "clutch_id.fct": the identity of the clutch attempt; "egg_id.scl.f": the egg position in the laying order scaled within clutches; "clutch_size.scl": the size of the clutch scaled and "tmin.mean.scl": the mean minimum temperature during the three days of egg formation scaled, "null" indicates a model with only the intercept, the mean in egg volume, "full", a model with all variables considered included.

Deviance clutch_volume

	model_type	null	ref	bigger	removed	null_deviance	ref_deviance
1	clutch_volume	null	null	null	null	0.00	0.00
2	clutch_volume	null	null	full	year.fct	7.20	7.20
3	clutch_volume	null	null	full	tmin.mean.scl	3.95	3.95
4	clutch_volume	null	null	full	clutch_size.scl	-0.06	-0.06

SI Table 13 | Evaluation of deviance explained per variable (clutch volume models): As per SI Table 12.

Deviance egg_width

	model_type	null	ref	bigger	removed	null_deviance	ref_deviance
1	egg_width	null	null	clutch_id.fct	clutch_id.fct	73.69	73.69
2	egg_width	null	clutch_id.fct	fullnoRS	fullRS	-10.41	-39.55
3	egg_width	null	clutch_id.fct	fullnoRS	year.fct	-0.00	-0.01
4	egg_width	null	clutch_id.fct	fullnoRS	egg_id.scl.f	6.75	25.64
5	egg_width	null	clutch_id.fct	fullnoRS	tmin.mean.scl	0.51	1.93
6	egg_width	null	clutch_id.fct	fullnoRS	clutch_size.scl	0.00	0.02
7	egg_width	null	clutch_id.fct	fullnoRS	egg_id.scl.f,clutch_size.scl	0.00	0.00
8	egg_width	null	clutch_id.fct	fullnoRS	tmin.mean.scl,clutch_size.scl	0.00	0.02
9	egg_width	null	clutch_id.fct	fullnoRS	egg_id.scl.f,tmin.mean.scl	0.21	0.78

SI Table 14 | Evaluation of deviance explained per variable (egg width models): As per SI Table 12.

Deviance egg_length

	model_type	null	ref	bigger	removed	null_deviance	ref_deviance
1	egg_length	null	null	clutch_id.fct	clutch_id.fct	78.21	78.21
2	egg_length	null	clutch_id.fct	fullnoRS	fullRS	-10.42	-47.83
3	egg_length	null	clutch_id.fct	fullnoRS	year.fct	-0.00	-0.00
4	egg_length	null	clutch_id.fct	fullnoRS	egg_id.scl.f	1.49	6.84
5	egg_length	null	clutch_id.fct	fullnoRS	tmin.mean.scl	0.21	0.97
6	egg_length	null	clutch_id.fct	fullnoRS	clutch_size.scl	0.10	0.47
7	egg_length	null	clutch_id.fct	fullnoRS	egg_id.scl.f,clutch_size.scl	0.10	0.46
8	egg_length	null	clutch_id.fct	fullnoRS	tmin.mean.scl,clutch_size.scl	-0.00	-0.00
9	egg_length	null	clutch_id.fct	fullnoRS	egg_id.scl.f,tmin.mean.scl	0.21	0.96

SI Table 15 | Evaluation of deviance explained per variable (egg length models): As per SI Table 12.

Deviance egg_sphericity

	model_type	null	ref	bigger	removed	null_deviance	ref_deviance
1	egg_sphericity	null	null	clutch_id.fct	clutch_id.fct	71.38	71.38
2	egg_sphericity	null	clutch_id.fct	fullnoRS	fullRS	-10.73	-37.48
3	egg_sphericity	null	clutch_id.fct	fullnoRS	year.fct	-0.00	-0.00
4	egg_sphericity	null	clutch_id.fct	fullnoRS	egg_id.scl.f	6.40	22.38
5	egg_sphericity	null	clutch_id.fct	fullnoRS	tmin.mean.scl	0.00	0.00
6	egg_sphericity	null	clutch_id.fct	fullnoRS	clutch_size.scl	0.14	0.51
7	egg_sphericity	null	clutch_id.fct	fullnoRS	egg_id.scl.f,clutch_size.scl	0.14	0.50
8	egg_sphericity	null	clutch_id.fct	fullnoRS	tmin.mean.scl,clutch_size.scl	-0.00	-0.00
9	egg_sphericity	null	clutch_id.fct	fullnoRS	egg_id.scl.f,tmin.mean.scl	-0.00	-0.00

SI Table 16 | Evaluation of deviance explained per variable (egg sphericity models): As per SI Table 12.

Variance

Variance egg_volume fullnoRS

	model	.component	.variance	.std_dev	.lower_ci	.upper_ci
1	egg_volume_04_fullnoRS	s(clutch_id.fct)	0.717	0.847	0.748	0.959
2	egg_volume_04_fullnoRS	s(year.fct)	0.027	0.165	0.037	0.736
3	egg_volume_04_fullnoRS	s(egg_id.scl.f)	0.146	0.382	0.132	1.105
4	egg_volume_04_fullnoRS	s(tmin.mean.scl)	0.000	0.000	0.000	3.067e+204
5	egg_volume_04_fullnoRS	s(clutch_size.scl)	0.000	0.000	0.000	Inf
6	egg_volume_04_fullnoRS	s(egg_id.scl.f,clutch_size.scl)	0.000	0.000	0.000	Inf
7	egg_volume_04_fullnoRS	s(tmin.mean.scl,clutch_size.scl)	0.000	0.000	0.000	Inf
8	egg_volume_04_fullnoRS	s(egg_id.scl.f,tmin.mean.scl)	0.128	0.358	0.114	1.124
9	egg_volume_04_fullnoRS	scale	0.216	0.465	0.444	0.487

SI Table 17 | Variance components (egg volume full model): Rows gather variables and columns the estimated variance components. Columns ".variance": estimated variance explained by each component, ".std_dev" the standard deviation, ".lower_ci" and ".upper_ci" the lower and upper bounds of the 95% confidence interval respectively. Rows gather the components in the model with variables included, "scale" being the remaining unexplained variation; **Random intercepts:** "year.fct": the year the breeding attempt took place, "clutch_id.fct" the identity of the clutch, **Main effects:** egg_id.scl.f": the egg position in the laying order scaled within clutches; "tmin.mean.scl": the mean minimum temperature during the three days of egg formation scaled, and "clutch_size.scl": the size of the clutch scaled and. **Interactions:** Any row with a combination of the above variables.

Variance clutch_volume full

	model	.component	.variance	.std_dev	.lower_ci	.upper_ci
1	clutch_volume_02_full	s(year.fct)	0.098	0.313	0.091	1.070
2	clutch_volume_02_full	s(tmin.mean.scl)	0.108	0.329	0.036	3.003
3	clutch_volume_02_full	s(clutch_size.scl)	0.000	0.003	0.000	4.000e+59
4	clutch_volume_02_full	scale	0.949	0.974	0.864	1.098

SI Table 18 | Variance components (clutch volume full model): As per SI table 17; **Random intercepts:** "year.fct": the year the breeding attempt took place, **Main effects:** "tmin.mean.scl": the mean minimum temperature during the three days of egg formation scaled, and "clutch_size.scl": the size of the clutch scaled.

Variance egg_width fullnoRS

	model	.component	.variance	.std_dev	.lower_ci	.upper_ci
1	egg_width_04_fullnoRS	s(clutch_id.fct)	0.665	0.815	0.720	0.924
2	egg_width_04_fullnoRS	s(year.fct)	0.033	0.182	0.043	0.763
3	egg_width_04_fullnoRS	s(egg_id.scl.f)	0.148	0.384	0.155	0.953
4	egg_width_04_fullnoRS	s(tmin.mean.scl)	0.075	0.273	0.052	1.431
5	egg_width_04_fullnoRS	s(clutch_size.scl)	0.000	0.001	0.000	Inf
6	egg_width_04_fullnoRS	s(egg_id.scl.f,clutch_size.scl)	0.000	0.000	0.000	Inf
7	egg_width_04_fullnoRS	s(tmin.mean.scl,clutch_size.scl)	0.009	0.094	0.000	4.261e+06
8	egg_width_04_fullnoRS	s(egg_id.scl.f,tmin.mean.scl)	0.140	0.374	0.096	1.455
9	egg_width_04_fullnoRS	scale	0.223	0.473	0.451	0.495

SI Table 19 | Variance components (egg width full model): As per SI table 17.

Variance egg_length fullnoRS

	model	.component	.variance	.std_dev	.lower_ci	.upper_ci
1	egg_length_04_fullnoRS	s(clutch_id.fct)	0.787	0.887	0.786	1.002
2	egg_length_04_fullnoRS	s(year.fct)	0.000	0.000	0.000	Inf
3	egg_length_04_fullnoRS	s(egg_id.scl.f)	0.262	0.511	0.166	1.573
4	egg_length_04_fullnoRS	s(tmin.mean.scl)	0.000	0.000	0.000	Inf
5	egg_length_04_fullnoRS	s(clutch_size.scl)	0.000	0.001	0.000	1.003e+205
6	egg_length_04_fullnoRS	s(egg_id.scl.f,clutch_size.scl)	0.063	0.251	0.049	1.297
7	egg_length_04_fullnoRS	s(tmin.mean.scl,clutch_size.scl)	0.000	0.000	0.000	Inf
8	egg_length_04_fullnoRS	s(egg_id.scl.f,tmin.mean.scl)	0.070	0.264	0.074	0.939
9	egg_length_04_fullnoRS	scale	0.234	0.484	0.462	0.506

SI Table 20 | Variance components (egg length full model): As per SI table 17.

Variance egg_sphericity fullnoRS

	model	.component	.variance	.std_dev	.lower_ci	.upper_ci
1	egg_sphericity_04_fullnoRS	s(clutch_id.fct)	0.722	0.849	0.752	0.960
2	egg_sphericity_04_fullnoRS	s(year.fct)	0.000	0.000	0.000	Inf
3	egg_sphericity_04_fullnoRS	s(egg_id.scl.f)	0.202	0.450	0.152	1.334
4	egg_sphericity_04_fullnoRS	s(tmin.mean.scl)	0.000	0.000	0.000	Inf
5	egg_sphericity_04_fullnoRS	s(clutch_size.scl)	0.000	0.000	0.000	Inf
6	egg_sphericity_04_fullnoRS	s(egg_id.scl.f,clutch_size.scl)	0.088	0.297	0.067	1.312
7	egg_sphericity_04_fullnoRS	s(tmin.mean.scl,clutch_size.scl)	0.000	0.000	0.000	1.016e+288
8	egg_sphericity_04_fullnoRS	s(egg_id.scl.f,tmin.mean.scl)	0.000	0.000	0.000	3.187e+248
9	egg_sphericity_04_fullnoRS	scale	0.255	0.505	0.482	0.528

SI Table 21 | Variance components (egg sphericity full model): As per SI table 17.

Significance tests for smooths

Significance egg_volume fullnoRS

	component	edf	Ref.df	F	p-value
1	s(clutch_id.fct)	133.456	143.000	19.583	0.000
2	s(year.fct)	1.849	4.000	90.942	0.278
3	s(egg_id.scl.f)	3.434	3.827	24.911	0.000
4	s(tmin.mean.scl)	1.000	1.000	0.483	0.487
5	s(clutch_size.scl)	1.000	1.000	0.978	0.323
6	s(egg_id.scl.f,clutch_size.scl)	0.000	2.000	0.000	0.410
7	s(tmin.mean.scl,clutch_size.scl)	0.000	2.000	0.000	0.676
8	s(egg_id.scl.f,tmin.mean.scl)	1.629	2.000	17.903	0.076

SI Table 22 | Significance for smooth terms (egg volume full model): Column "component": the name of each smooth term **Random intercepts:** "year.fct": the year the breeding attempt took place, "clutch_id.fct" the identity of the clutch, **Main effects:** egg_id.scl.f": the egg position in the laying order scaled within clutches; "tmin.mean.scl": the mean minimum temperature during the three days of egg formation scaled, and "clutch_size.scl" : the size of the clutch scaled and. **Interactions:** Any row with a combination of the above variables. Columns "edf" indicate the estimated degrees of freedom after shrinkage through penalized log-likelihood on the wiggliness of smooth function, "Ref.df" the user-defined degrees of freedom on which shrinkage acted, "F" the statistic summarizing the variance used together with the Ref.df value in Wald-like test of the null hypothesis that the smooth function is a constant flat function from which the probability is obtained "p-value" (see Wood 2017 section 6.12).

Significance clutch_volume full

	component	edf	Ref.df	F	p-value
1	s(year.fct)	2.630	4.000	1.687	0.040
2	s(tmin.mean.scl)	1.793	2.242	1.016	0.365
3	s(clutch_size.scl)	1.000	1.000	0.636	0.426

SI Table 23 | Significance for smooth terms (clutch volume full model): As per SI table 22.

Significance egg_width fullnoRS

	component	edf	Ref.df	F	p-value
1	s(clutch_id.fct)	132.565	143.000	17.916	0.000
2	s(year.fct)	2.081	4.000	106.618	0.188
3	s(egg_id.scl.f)	3.397	3.789	66.310	0.000
4	s(tmin.mean.scl)	3.518	4.170	1.161	0.513
5	s(clutch_size.scl)	1.000	1.000	0.401	0.527
6	s(egg_id.scl.f,clutch_size.scl)	0.000	2.000	0.000	0.661
7	s(tmin.mean.scl,clutch_size.scl)	0.101	2.000	0.417	0.258
8	s(egg_id.scl.f,tmin.mean.scl)	1.100	2.000	8.739	0.002

SI Table 23 | Significance for smooth terms (egg width full model): As per SI Table 22.

Significance egg_length fullnoRS

	component	edf	Ref.df	F	p-value
1	s(clutch_id.fct)	136.151	143.000	20.340	0.000
2	s(year.fct)	0.000	4.000	0.000	0.512
3	s(egg_id.scl.f)	3.595	3.903	11.947	0.000
4	s(tmin.mean.scl)	1.000	1.000	0.042	0.838
5	s(clutch_size.scl)	1.000	1.000	0.923	0.337
6	s(egg_id.scl.f,clutch_size.scl)	0.895	2.000	2.676	0.521
7	s(tmin.mean.scl,clutch_size.scl)	0.000	2.000	0.000	0.956
8	s(egg_id.scl.f,tmin.mean.scl)	1.409	2.000	8.177	0.212

SI Table 24 | Significance for smooth terms (egg length full model): As per SI Table 22.

Significance egg_sphericity fullnoRS

	component	edf	Ref.df	F	p-value
1	s(clutch_id.fct)	135.661	143.000	18.961	0.000
2	s(year.fct)	0.000	4.000	0.000	0.629
3	s(egg_id.scl.f)	3.479	3.843	67.063	0.000
4	s(tmin.mean.scl)	1.000	1.000	0.000	0.995
5	s(clutch_size.scl)	1.000	1.000	0.110	0.740
6	s(egg_id.scl.f,clutch_size.scl)	1.020	2.000	5.143	0.478
7	s(tmin.mean.scl,clutch_size.scl)	0.000	2.000	0.000	0.906
8	s(egg_id.scl.f,tmin.mean.scl)	0.000	2.000	0.000	0.879

SI Table 25 | Significance for smooth terms (egg sphericity full model): As per SI Table 22.

Effects covariates - Egg volume full model

Random effects

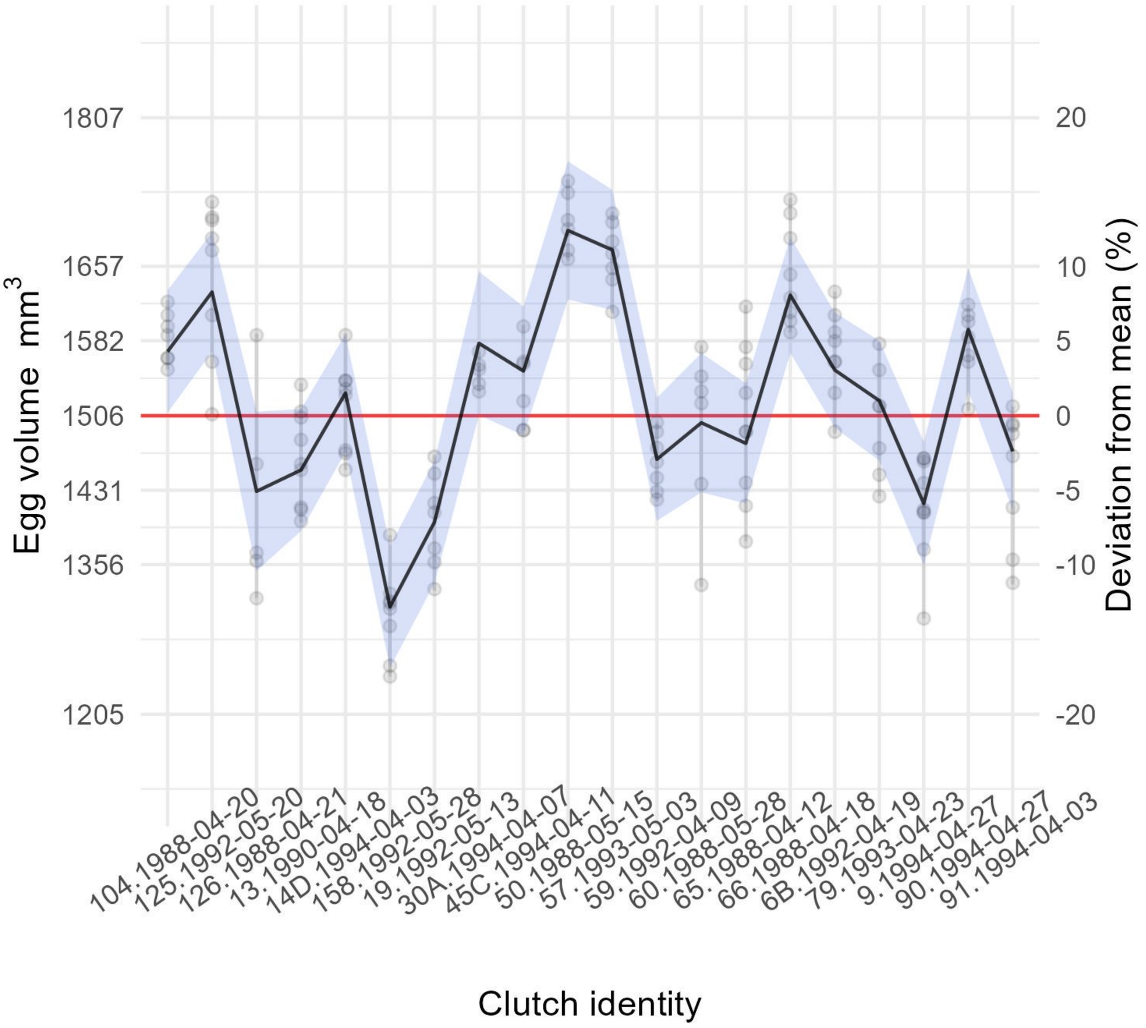

SI Figure 10 | Egg volume as a function of clutch identity (egg volume full model): The x axis shows a random sample of 20 clutches. The y axis on the left shows the egg volume in mm^3 while the y axis on the right represents egg volumes as a deviation in percentage from the mean egg volume. The points show values observed and grey lines connect values from eggs in the same clutch. The red line indicates the mean egg volume observed. The black line indicates the predicted values by the GAM model excluding the effects of other variables with the 95% confidence interval in shaded blue.

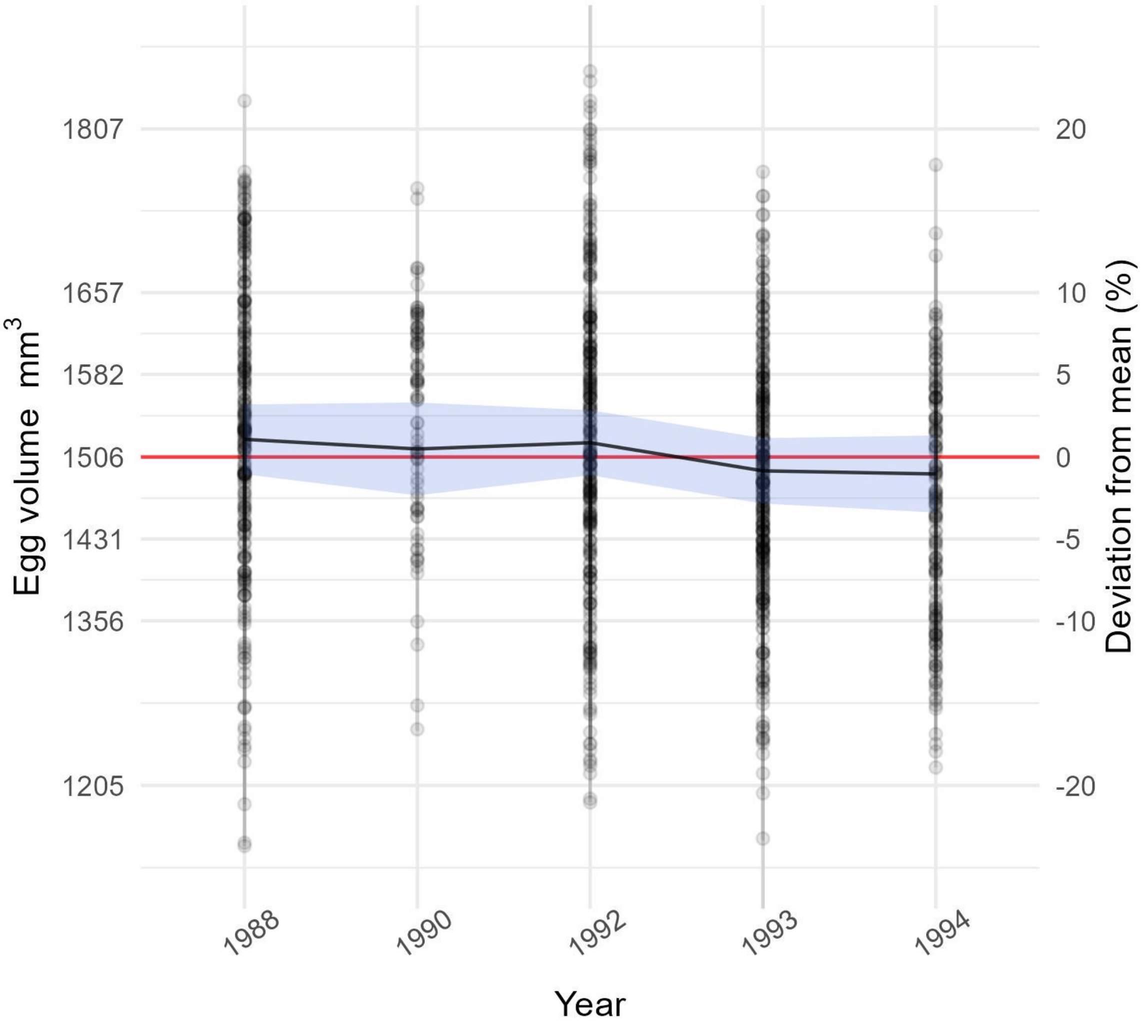

SI Figure 11 | Egg volume as a function of year (egg volume full model): The x axis shows the five years of data collection. The y axis on the left shows the egg volume in mm^3 while the y axis on the right represents egg volumes as a deviation in percentage from the mean egg volume. The points show values observed and grey lines connect values from eggs in the same clutch. The red line indicates the mean egg volume observed. The black line indicates the predicted values by the GAM model excluding the effects of other variables with the 95% confidence interval in shaded blue.

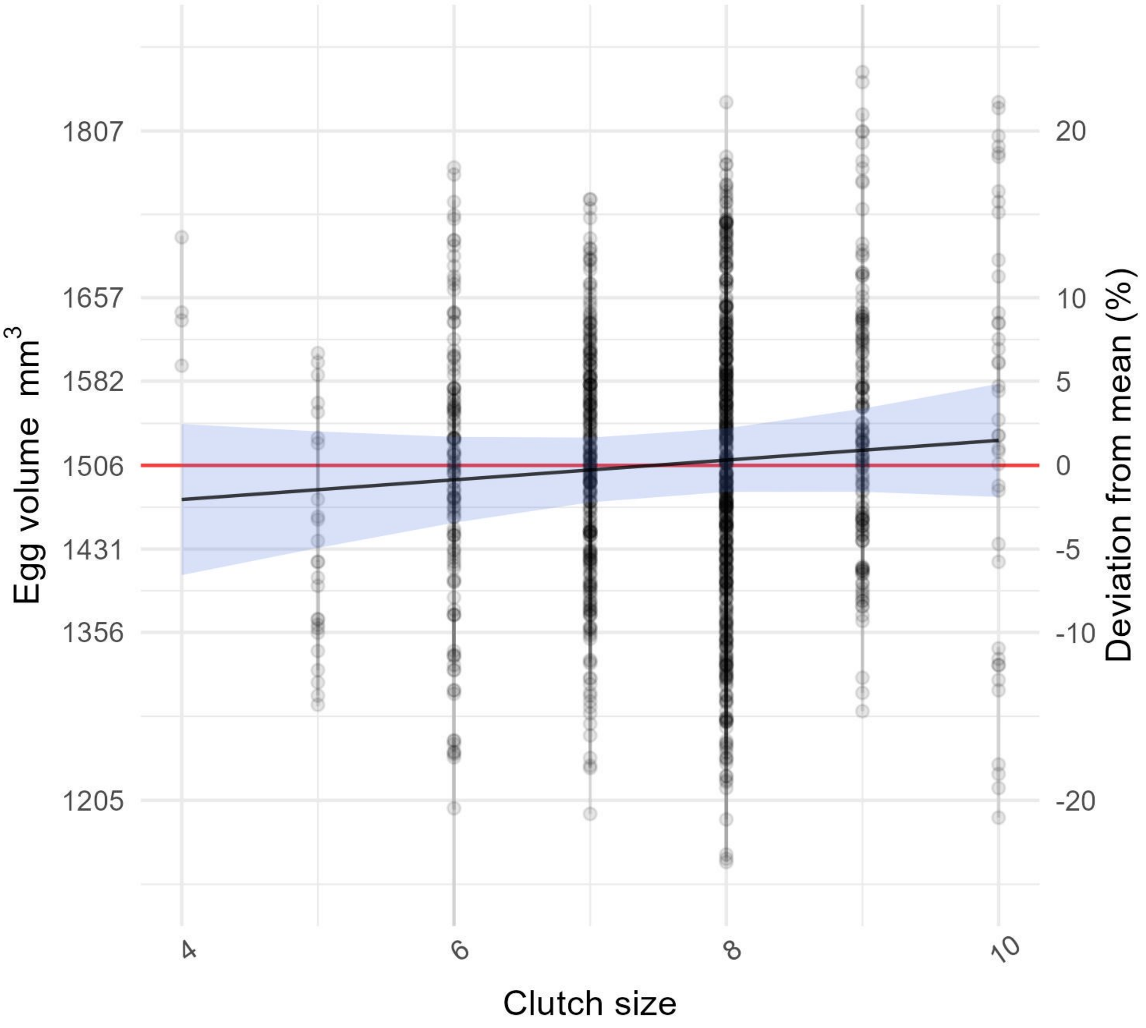

SI Figure 12 | Egg volume as a function of clutch size (egg volume full model): The x axis shows the total clutch size, y axis on the left shows the egg volume in mm^3, while the y axis on the right represents egg volumes as a deviation in percentage from the mean egg volume. The points show values observed and grey lines connect values from eggs in the same clutch. The red line indicates the mean egg volume observed. The black line indicates the predicted values by the GAM model excluding the effects of other variables with the 95% confidence interval in shaded blue.

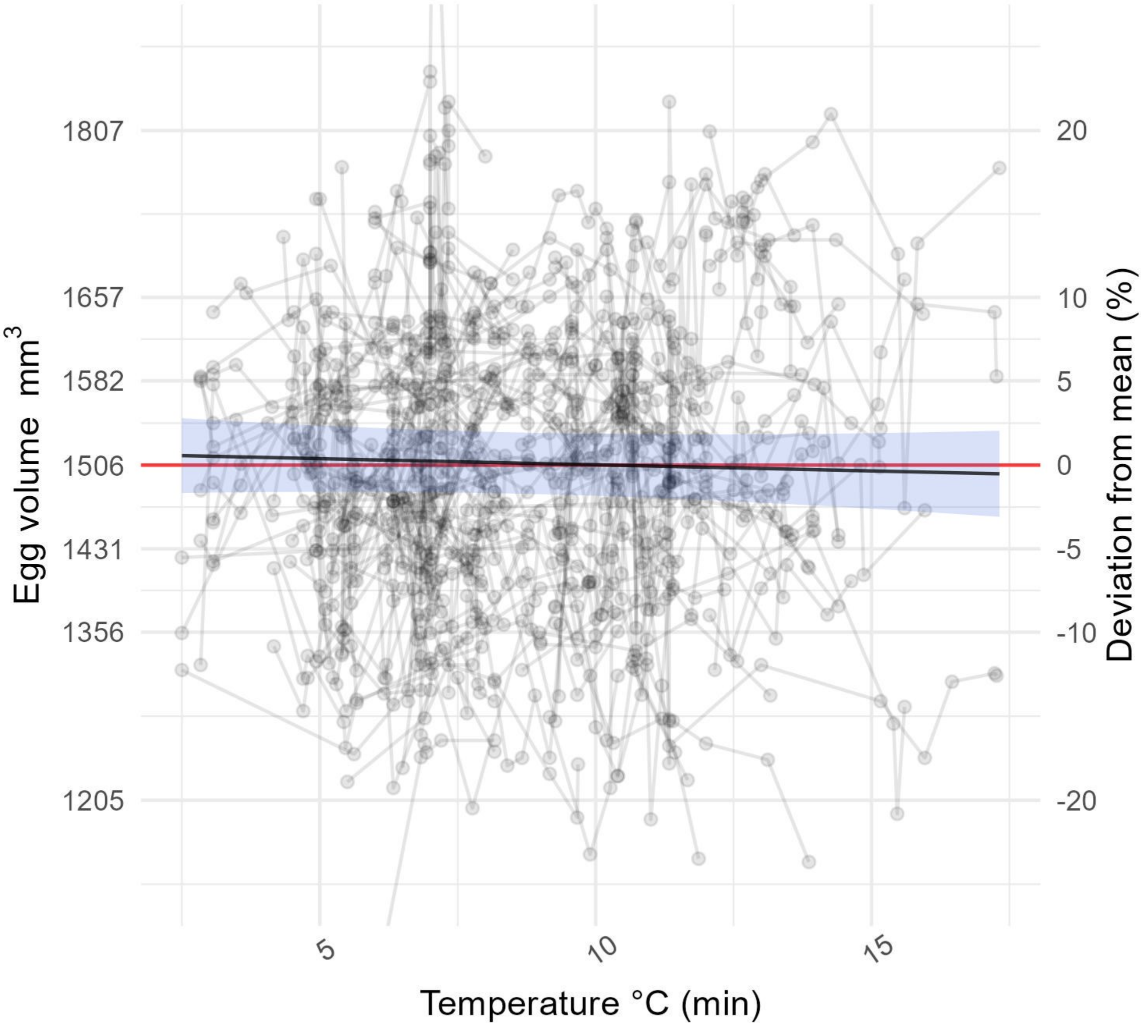

SI Figure 13 | Egg volume as a function of minimum temperatures (egg volume full model): The x axis shows the mean minimum temperature in °C for the three days of egg formation, y axis on the left shows the egg volume in mm^3 while the y axis on the right represents the egg volumes as deviation in percentage from the mean egg volume. The points show values observed, and grey lines connect values from eggs in the same clutch. The red line indicates the mean egg volume observed. The black line indicates the predicted values by the GAM model excluding effects of other variables with the 95% confidence interval in shaded blue.

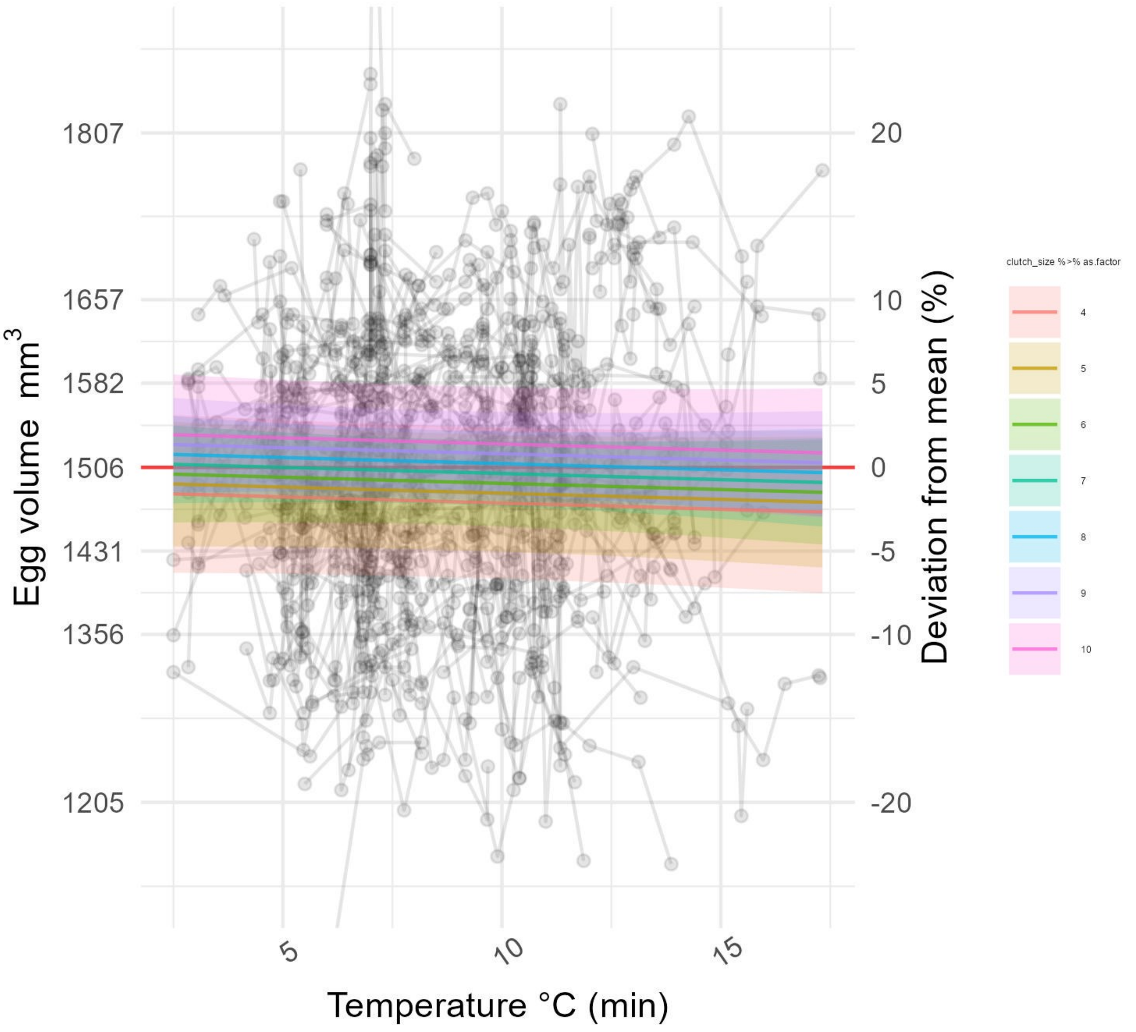

SI Figure 14 | Egg volume as a function of minimum temperatures per clutch size (egg volume full model): The x axis shows the mean minimum temperature in °C for the three days of egg formation, y axis on the left shows the egg volume in mm^3 while the y axis on the right represents egg volumes as a deviation in percentage from the mean egg volume. The points show values observed and grey lines connect values from eggs in the same clutch. The red line indicates the mean egg volume observed. The colored lines with shaded areas indicate the predicted values and 95% confidence intervals, respectively, generated by the GAM model when excluding all other variables' effects and leaving the main effects and their interaction.

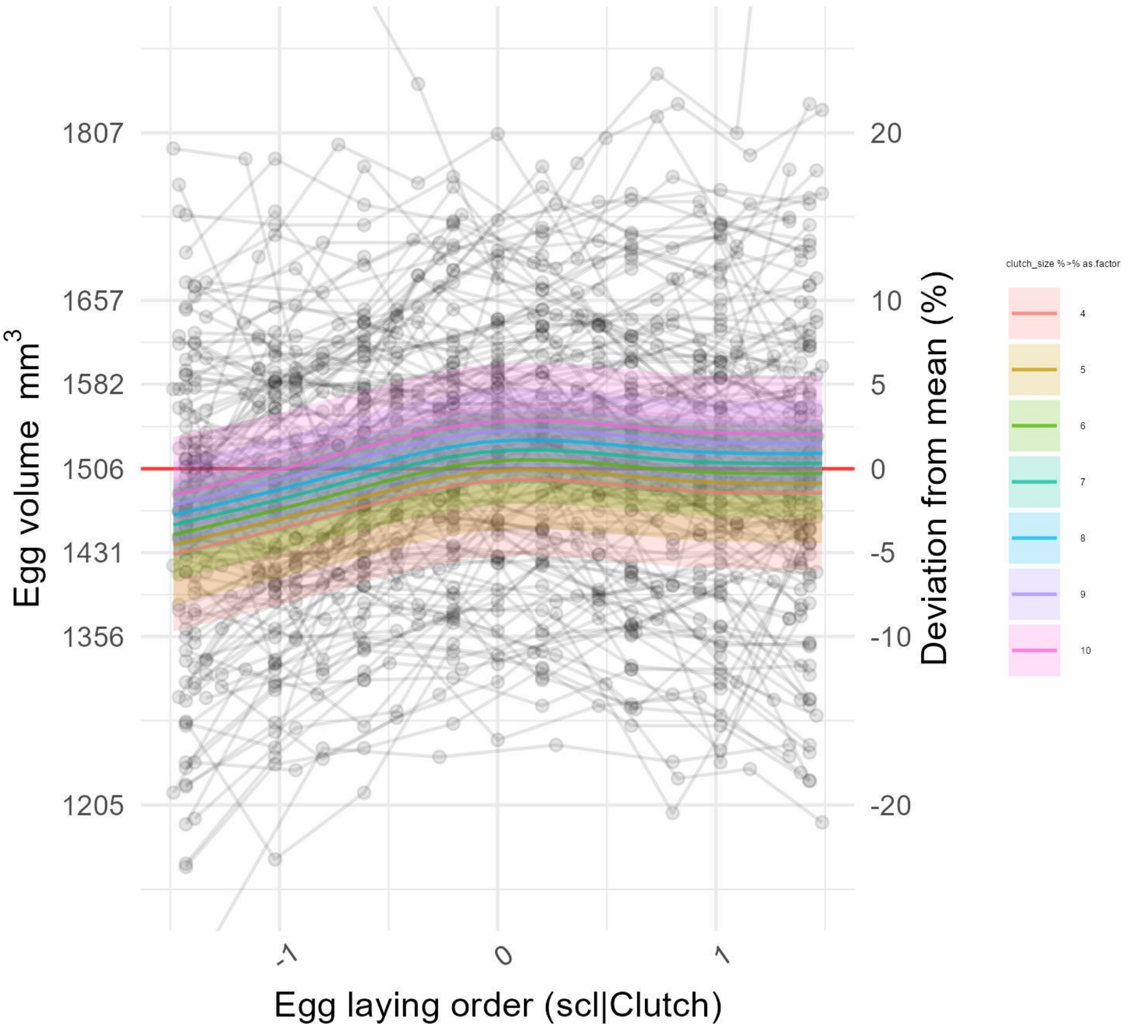

SI Figure 15 | Egg volume as a function of laying order per clutch size (egg volume full model): The x axis shows the position in the egg laying order of a given egg scaled per clutch size, y axis on the left shows the egg volume in mm^3 while the y axis on the right represents egg volumes as deviation in percentage from the mean egg volume. The points show values observed and grey lines connect values from eggs in the same clutch. The red line indicates the mean egg volume observed. The colored lines with shaded areas indicating the predicted values and 95% confidence intervals respectively generated by the GAM model when excluding all other variables' effects and leaving the main effects and their interaction.

Autocorrelation

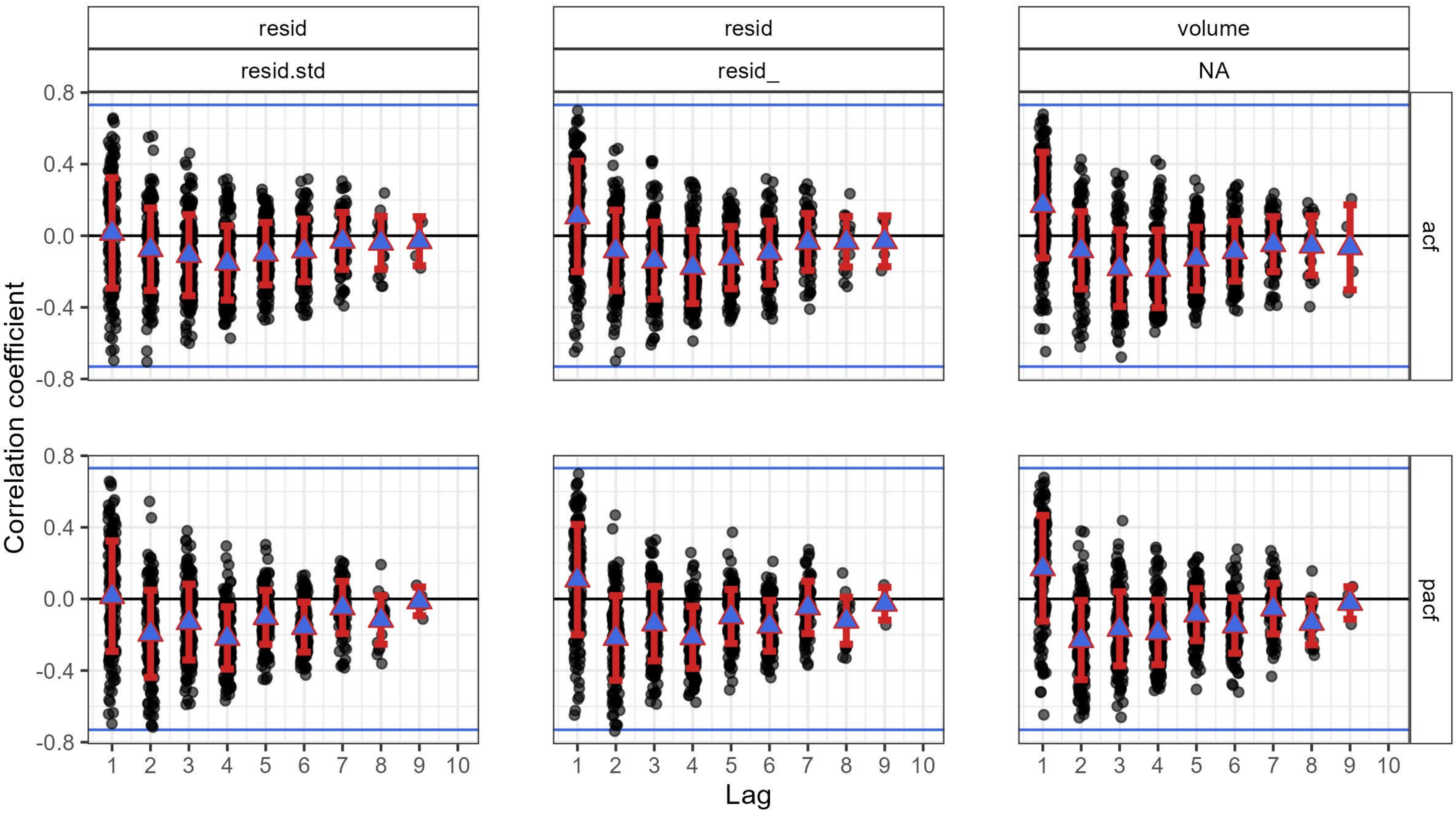

SI Figure 16 | Autocorrelation coefficients for residuals (egg volume full model): The x axis shows the days of lag between two egg values. Y axis shows the strength of the correlation coefficient. Points indicate calculated values for a given clutch, blue triangles the mean for a given lag with standard deviations as arms. The panels are structured in rows containing measures from autocorrelation and partial autocorrelation function in the upper and lower row, respectively. Columns indicate the variable with left the standardized residuals with the AR1 errors, middle the raw residuals and right the egg volume scaled. The black lines indicate the zero value of correlation, blue lines indicate the correlation values for which a random noise series could fall for the sample size of the mean clutch size.

Effects covariates - Clutch full model

Random effects

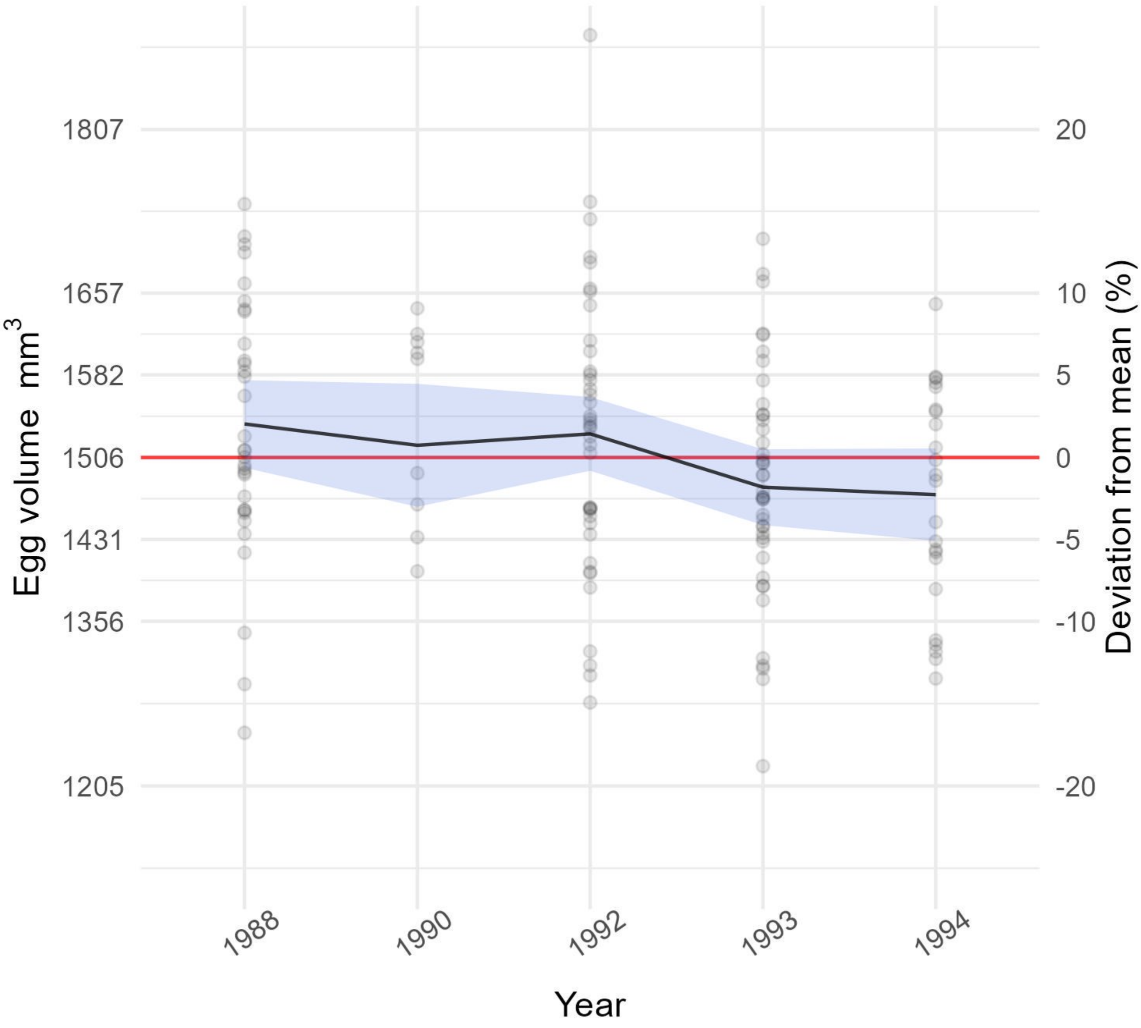

SI Figure 17 | Egg volume as a function of year (clutch volume full model): The x axis shows the five years of data collection. The y axis on the left shows the egg volume in mm^3 while the y axis on the right represents egg volumes as a deviation in percentage from the mean egg volume. The points show values observed. The red line indicates the mean egg volume observed. The black line indicates the predicted values by the GAM model, excluding the effects of other variables, with the 95% confidence interval in shaded blue.

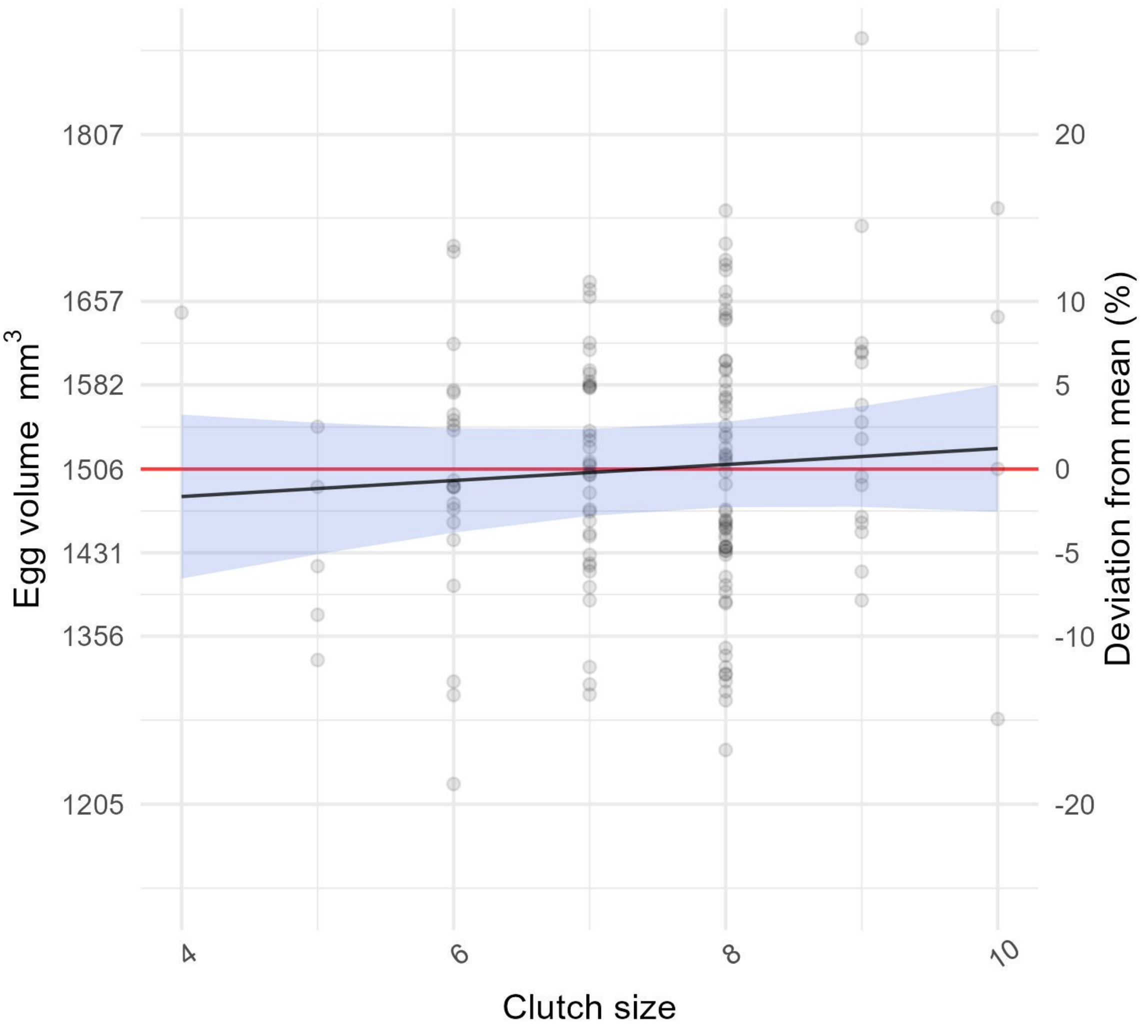

SI Figure 18 | Egg volume as a function of clutch size (clutch volume full model): The x axis shows the total clutch size, y axis on the left shows the egg volume in mm^3 while the y axis on the right represents egg volumes as a deviation in percentage from the mean egg volume. The points show values observed. The red line indicates the mean egg volume observed. The black line indicates the predicted values by the GAM model, excluding the effects of other variables, with the 95% confidence interval in shaded blue.

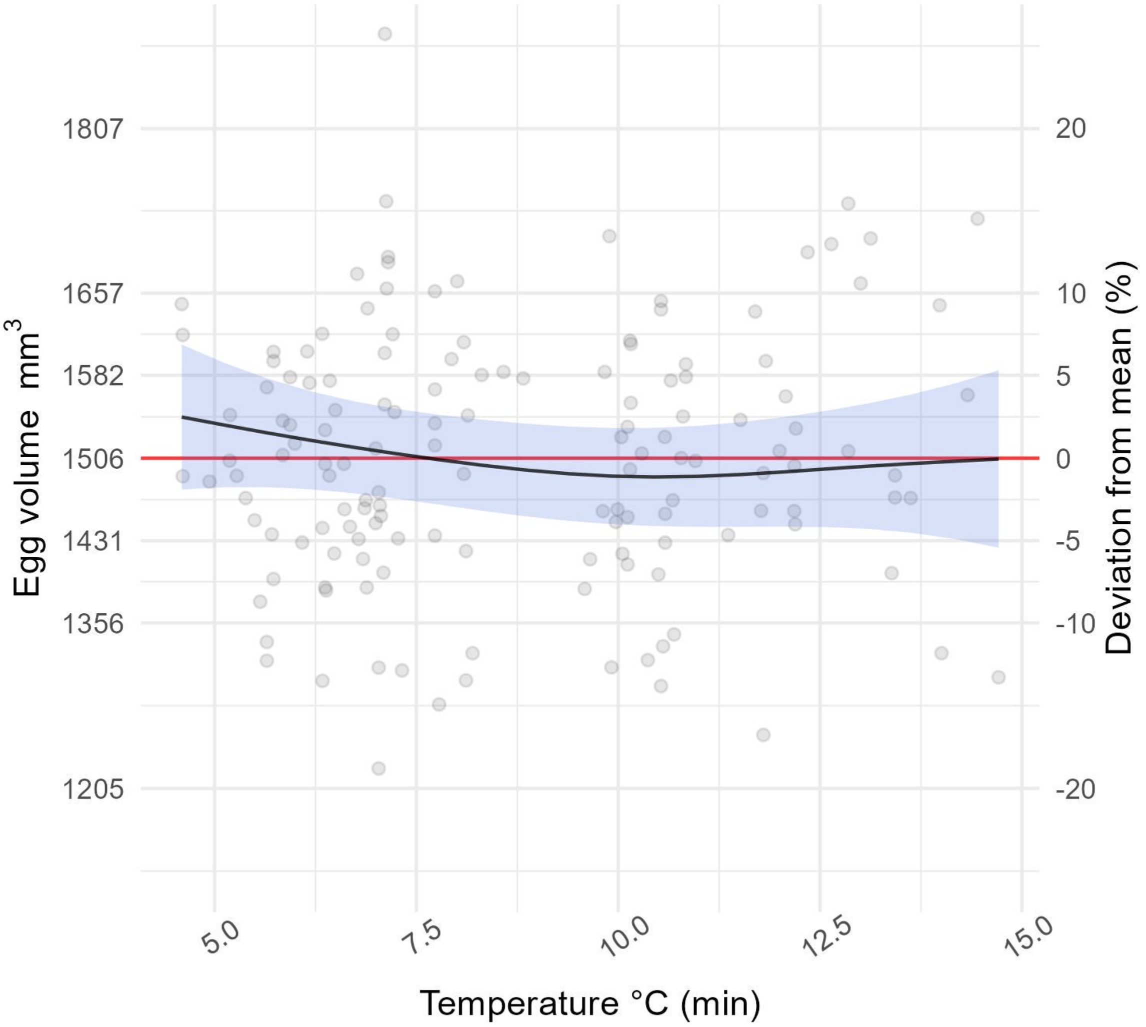

SI Figure 19 | Egg volume as a function of minimum temperatures (clutch volume full model): The x axis shows the mean minimum temperature in °C for the three days of egg formation, y axis on the left shows the mean egg volume per clutch in mm^3 while the y axis on the right represents mean egg volumes per clutch as a deviation in percentage from the mean egg volume. The points show values observed. The red line indicates the mean egg volume observed. The black line indicates the predicted values by the GAM model, excluding the effects of other variables, with the 95% confidence interval in shaded blue.

Effects covariates - Egg width length sphericity full models

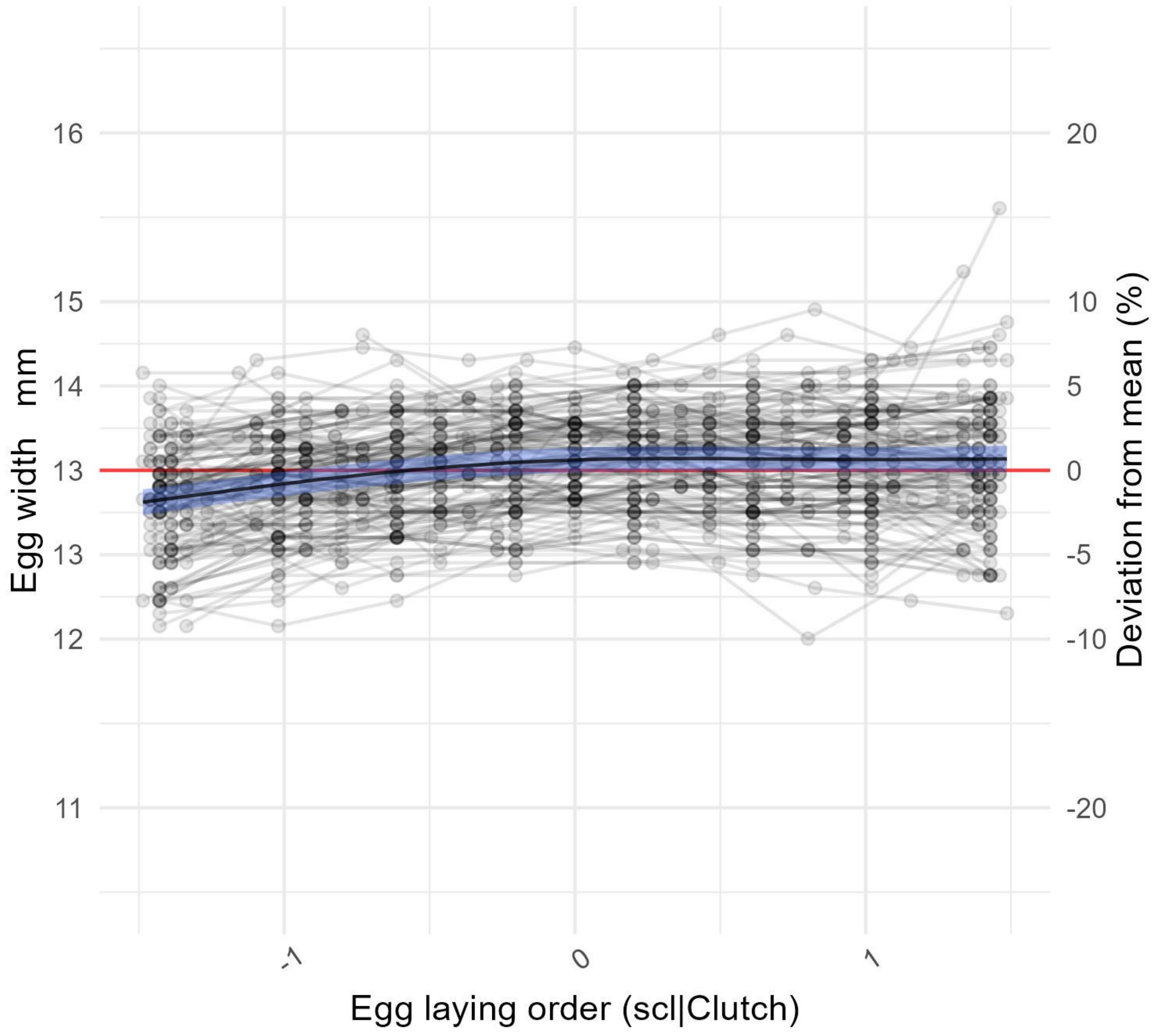

SI Figure 20 | Egg width as a function of laying order: The x axis shows the position in the egg laying order of a given egg scaled per clutch size, y axis on the left shows the egg width in mm while the y axis on the right represents egg width as deviation in percentage from the mean egg width. The points show values observed and grey lines connect values from eggs in the same brood. The red line indicates the mean egg volume observed. The black line indicates the predicted values by the GAMM model excluding effects of other variables with the 95% confidence interval in shaded blue.

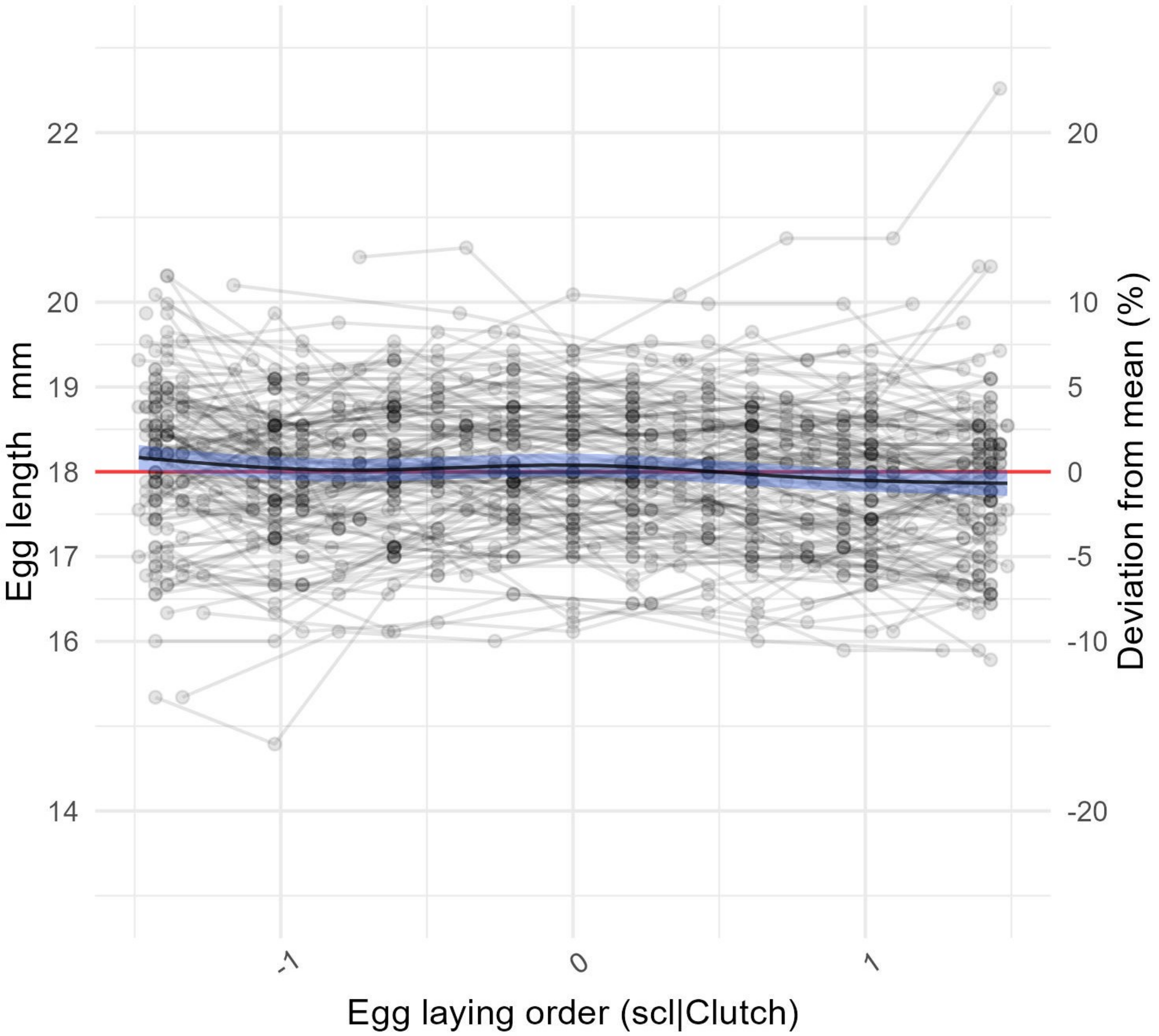

SI Figure 21 | Egg length as a function of laying order: The x axis shows the position in the egg laying order of a given egg scaled per clutch size, y axis on the left shows the egg length in mm while the y axis on the right represents egg length as deviation in percentage from the mean egg length. The points show values observed and grey lines connect values from eggs in the same brood. The red line indicates the mean egg volume observed. The black line indicates the predicted values by the GAMM model excluding effects of other variables with the 95% confidence interval in shaded blue.

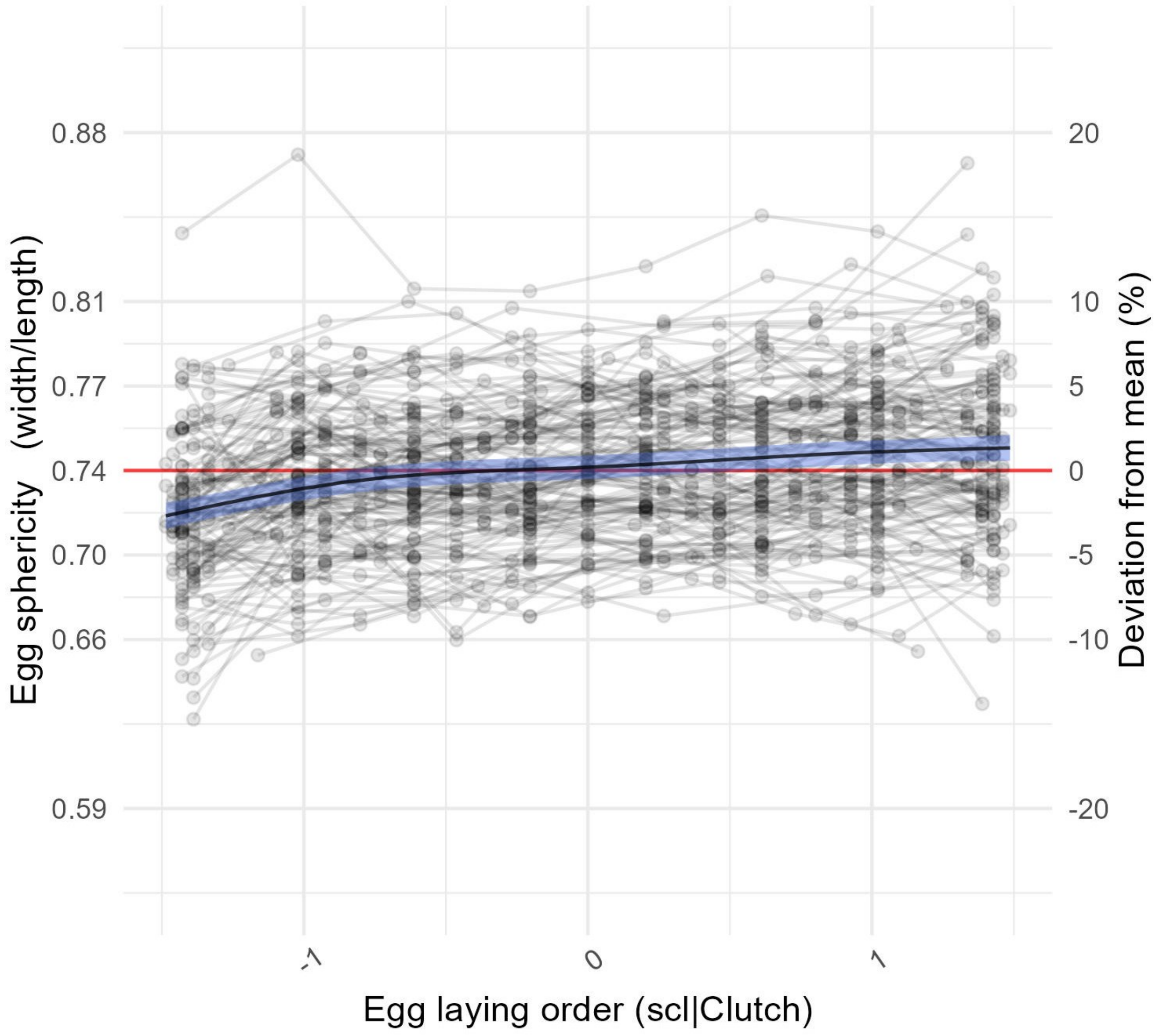

SI Figure 22 | Egg sphericity as a function of laying order: The x axis shows the position in the egg laying order of a given egg scaled per clutch size, y axis on the left shows the egg sphericity in proportion while the y axis on the right represents egg sphericity as deviation in percentage from the mean egg sphericity. The points show values observed and grey lines connect values from eggs in the same brood. The red line indicates the mean egg volume observed. The black line indicates the predicted values by the GAMM model excluding effects of other variables with the 95% confidence

R Packages

Package tidyverse (Wickham et al. 2019) was used to implement code based on subpackages dplyr, purrr, ggplot2 and lubridate. Further manipulation of data was made available by using package stringi (Gagolewski 2022). Packages knitr (Xie 2023) and gridExtra (Auguie 2017) were used for exporting tables. Package patchwork (Pedersen 2024) was used to arrange plots. Packages plotly (Sievert 2020) and reticulate (Ushey et al. 2023) were used to produce and export 3d plots via Python software (Van Rossum and Drake Jr 1995). Package Hmisc (Harrell Jr 2025) was used for plots regarding autocorrelation. Package mgcv (Wood 2017) and gratia (Simpson 2024) were used to fit, diagnose and predict from GAMMs.

Bibliography

Auguie B (2017) gridExtra: Miscellaneous Functions for “Grid” Graphics

Gagolewski M (2022) stringi: Fast and portable character string processing in R. Journal of Statistical Software 103:1–59. https://doi.org/10.18637/jss.v103.i02

Harrell Jr FE (2025) Hmisc: Harrell miscellaneous

Pedersen TL (2024) patchwork: The composer of plots

Sievert C (2020) Interactive web-based data visualization with R, plotly, and shiny. Chapman and Hall/CRC

Simpson GL (2024) gratia: Graceful ggplo-based graphics and other functions for GAMs fitted using mgcv

Ushey K, Allaire J, Tang Y (2023) reticulate: Interface to “Python”

Van Rossum G, Drake Jr FL (1995) Python tutorial. Centrum voor Wiskunde en Informatica Amsterdam, The Netherlands

Wickham H, Averick M, Bryan J, et al (2019) Welcome to the tidyverse. Journal of Open Source Software 4:1686. https://doi.org/10.21105/joss.01686

Wood SN (2017) Generalized Additive Models: An Introduction with R, Second Edition, 2nd edition. Taylor & Francis Inc, Boca Raton

Xie Y (2023) knitr: a general-purpose package for dynamic report generation in R
